# Evidence That Increasing Serum 25(OH)D Concentrations to 30 ng/mL in the Kingdom of Saudi Arabia and the United Arab Emirates Could Greatly Improve Health Outcomes

**DOI:** 10.3390/biomedicines11040994

**Published:** 2023-03-23

**Authors:** William B. Grant, Fatme Al Anouti, Barbara J. Boucher, Hana M. A. Fakhoury, Meis Moukayed, Stefan Pilz, Nasser M. Al-Daghri

**Affiliations:** 1 Sunlight, Nutrition, and Health Research Center, P.O. Box 641603, San Francisco, CA 94164-1603, USA; 2 Department of Health Sciences, College of Natural and Health Sciences, Zayed University, Abu Dhabi P.O. Box 144534, United Arab Emirates; 3 The Blizard Institute, Barts & The London School of Medicine & Dentistry, Queen Mary University of London, London E12AT, UK; 4 Department of Biochemistry and Molecular Medicine, College of Medicine, Alfaisal University, Riyadh 11533, Saudi Arabia; 5 School of Arts and Sciences, American University in Dubai, Dubai P.O. Box 28282, United Arab Emirates; 6 Division of Endocrinology and Diabetology, Department of Internal Medicine, Medical University of Graz, 8036 Graz, Austria; 7 Chair for Biomarkers of Chronic Diseases, Biochemistry Department, College of Science, King Saud University, Riyadh 11451, Saudi Arabia

**Keywords:** breast cancer, calcitriol, case-control study, dementia, hill’s criteria, metabolic syndrome, Middle East, recommendations, season, solar UVB

## Abstract

Accumulating evidence supports the potential protective effects of vitamin D against chronic diseases such as Alzheimer’s disease, autoimmune diseases, cancers, cardiovascular disease (ischaemic heart disease and stroke), type 2 diabetes, hypertension, chronic kidney disease, stroke, and infectious diseases such as acute respiratory tract diseases, COVID-19, influenza, and pneumonia, as well as adverse pregnancy outcomes. The respective evidence is based on ecological and observational studies, randomized controlled trials, mechanistic studies, and Mendelian randomization studies. However, randomized controlled trials on vitamin D supplementation have largely failed to show benefits, probably due to poor design and analysis. In this work, we aim to use the best available evidence on the potential beneficial effects of vitamin D to estimate the expected reduction in incidence and mortality rates of vitamin D-related diseases in the Kingdom of Saudi Arabia and the United Arab Emirates if minimum serum 25(OH)D concentrations were to be raised to 30 ng/mL. Estimated reductions by 25% for myocardial infarction incidence, 35% for stroke incidence, 20 to 35% for cardiovascular disease mortality, and 35% for cancer mortality rates depicted a promising potential for raising serum 25(OH)D. Methods to increase serum 25(OH)D concentrations at the population level could include food fortification with vitamin D_3_, vitamin D supplementation, improved dietary vitamin D intake, and sensible sun exposure.

## 1. Introduction

This is the centennial of the naming of vitamin D by Elmer McCollum et al. in 1922 [1]. For most of the last 100 years, vitamin D has been best known for its classical effects on regulation of calcium and phosphorus absorption from the gastrointestinal tract as well as their metabolism in relation to bone health [2]. In view of those effects, the Institute of Medicine recommends 600 to 800 IU/day vitamin D supplementation to achieve a 25-hydroxyvitamin D [25(OH)D] concentration of 20 ng/mL (50 nmol/L) [3] while the U.S. Endocrine Society recommends 1500–2000 IU/d vitamin D supplementation for those with vitamin D deficiency (<20 ng/mL) to achieve a 25(OH)D value > 30 ng/mL [4].

National vitamin D guidelines have been in place in the Kingdom of Saudi Arabia (KSA) as well as for the Gulf Cooperating Council (GCC) countries, a regional union of Arab monarchies that include not only KSA and UAE, but also for Bahrain, Kuwait, Oman and Qatar since 2000. Both vitamin D guidelines advocated 25(OH)D cut-offs on the basis of bone health, and achieving ≥30 ng/mL (75 nmol/L) is currently only advised for the osteoporotic elderly and frail [5,6]. However, no consideration was given to younger vitamin D-deficient individuals with metabolic diseases, given the lack of local and regional data at the time of drafting the guidelines. Thus, these individuals are managed in the same way as the general population, that is, with the aim of achieving 25(OH)D ≥ 20 ng/mL (50 nmol/L), as advised [5,6]. Future guidelines should take into consideration the value of expanding the goal to cover the entire population and not just the elderly and frail.

Numerous potential non-skeletal (i.e., pleiotropic) effects for vitamin D have been intensively studied during the past two decades, but the first suggestion that vitamin D might play important roles in reducing extra-skeletal disease risks was made much earlier than that. A geographical ecological study in 1980 suggested that solar radiation [through production of vitamin D] reduced colon cancer mortality rates [7]. This was followed by finding that vitamin D was necessary for normal insulin secretion (e.g., [8]), by an observational study of dietary vitamin D and calcium intakes and the risks of colon cancer in 1985 [9], and of 25(OH)D concentrations in relation to the risks of colon cancer in 1989 [10]. A temporal ecological study in 1981 was used to suggest that solar UVB, through production of vitamin D_3_, reduced the risk of myocardial infarction (MI) [11], followed by an observational study of 25(OH)D and MI risks in 1990 [12]. A similar ecological study suggested that higher vitamin D status reduced the risk of epidemic influenza [13]. That study was quickly reinforced by a secondary analysis of a vitamin D randomized controlled trial (RCT) [14]. Studies such as a vitamin D RCT for cancer in 2007 [15], an observational study of 25(OH)D concentration and cardiovascular disease incidence in 2008 [16], and an RCT for vitamin D and influenza in 2011 provided more support for potential extra-skeletal benefits of vitamin D [17].

The pathophysiological mechanisms through which vitamin D may reduce the risk of various diseases have also been studied, e.g., [18,19]. Calcitriol—activated vitamin D produced in target tissues—enhances effects that protect the tissues in various ways which include the following: combating microbial and viral infections (innate immunity) by increasing the secretion of antimicrobials (e.g., cathelicidin and the two defensins [20]); acquired immunity is modulated to protect against excessive inflammatory damage and cytokine storms by the down-regulation of pro-inflammatory cytokines and the upregulation of anti-inflammatory cytokines [21]. Insulin resistance is combated by reducing the secretion of cell-damaging free radicals and UCP2 in hepatocytes, and by reducing FOXO1 secretion in muscle cells [22,23,24]; the secretion of the matrix metalloproteinases, 2/9 in particular, that lead to the inflammatory tissue destruction in arterial plaque that precipitates acute arterial events, to tissue caseation (liquifaction) in tuberculosis, causing lung cavitation, cartilage destruction and other joint damage in inflammatory arthritis, is strikingly reduced by the correction of vitamin D deficiency [25,26]. Further beneficial effects are being reported on neurological tissues, in part at least through effects on Wnt/β-catenin and the Sonic Hedgehog pathway modulation of neurogenesis [27]. Vitamin D may reduce the risk of cancer incidence through controlling differentiation, proliferation, and apoptosis of cells, and reduces cancer mortality risks through antiangiogenesis around tumors and through reducing the development of metastasis [28].

Based on the advances in understanding the role of vitamin D for non-skeletal diseases, some recommendations were made to increase serum 25(OH)D concentrations to 30–50 ng/mL (multiply ng/mL by 2.496 to convert ng/mL to nmol/L) [29,30]; however, recommendations by others have remained at 20 ng/mL, largely because RCTs have been unable to find beneficial effects of vitamin D supplementation and due to neglecting the findings from Mendelian randomization (MR) studies [31] and ignoring the limitations of vitamin D RCTs. For example, vitamin D RCTs have usually been based on guidelines for pharmaceutical drugs, but they should be based on guidelines for nutrients as outlined by Heaney [32,33]. The difference is that the drug guidelines are usually analysed by dosages of drug intakes while nutrient guidelines are based on baseline and achieved nutrient status (serum 25(OH)D concentrations in the case of vitamin D). There are also large interpersonal differences in baseline 25(OH)D concentrations and in the increases in 25(OH)D concentrations in response to any given vitamin D dose, such that the vitamin D dose in an RCT may be of little relevance for analyzing the results of supplementation. A prime example comes from the D2d trial that examined progression from prediabetes to diabetes [34]. When analyzed according to intention to treat, i.e., by dose, there was no significant difference between the outcomes for the vitamin D treatment and placebo arms. However, when the results were analyzed according to achieved 25(OH)D concentrations, it was found that for each 10 ng/mL increase in serum 25(OH)D of up to >50 ng/mL on vitamin D supplementation, there was a ten percent reduction in progression to diabetes which provided reductions of up to 70% in treated subjects achieving serum 25(OH)D values of ≥40 ng/mL [35].

Another way to evaluate whether vitamin D affects the risk of adverse health outcomes is through the use of Hill’s criteria for causality in a biological system [36]. The suitable criteria for evaluating vitamin D would encompass factors such as the strength of association, consistency, temporal relationship, biological gradient, feasibility of mechanisms, consistency with established facts regarding the disease natural history and biology, experimental evidence, and comparison to similar situations. Hill also stated that not all criteria need to be satisfied in order for causality to be claimed, but the more that are, the better. As will be discussed, observational studies generally find significant inverse correlations between serum 25(OH)D concentrations and adverse health outcomes if the reduction in association strength due to changes in serum 25(OH)D concentrations during long follow-up times is taken into account. Combining such studies in meta-analyses may allow observational studies to be used to estimate the effects on adverse health outcomes. The well-known mechanisms whereby vitamin D reduces adverse health outcomes are denoted in this article.

MR studies are increasingly being used to examine whether higher vitamin D status reduces the incidence of, or mortality from, various diseases. These analyses use data from large cohorts of subjects and are considered to be as reliable as adequately conducted randomized controlled trials [37]. Now that data from large representative cohorts from various populations are becoming available, the findings from MR studies are of increasing importance. MR studies examine the correlation between genetically-determined variation in 25(OH)D concentrations and health outcomes. There are many different genes involved in the vitamin D pathway, from those affecting vitamin D_3_ production in the skin to those producing 25(OH)D from vitamin D in the liver, those varying the binding of 25(OH)D to circulating proteins, and those activating 25(OH)D to produce the hormonally active metabolite calcitriol in all target tissues. Specific alleles of many of these genes are associated with consistent variation in serum 25(OH)D concentrations. Large databases of data for such variance are used to generate estimates of serum 25(OH)D variance with such polymorphisms and the associations of those polymorphisms with health risks are then used as a surrogate for the life-long variation of vitamin D status.

The use of genetically predicted 25(OH)D concentrations in MR effectively randomizes the participants into the study groups since this form of allocation is independent of such factors as vitamin D supplementation and solar UVB exposure. Since, however, increases in serum 25(OH)D produce benefits which plateau observationally once serum 25(OH)D levels reach a certain level, as for all nutrients, it is not surprising that many MR analyses have failed to show variance in health benefits using genome-wide association study (GWAS) data for all subjects. However, when GWAS data is used in subjects whose serum 25(OH)D values lie below the plateau value of 25(OH)D (i.e., the value above which the health benefit of interest ceases to increase in observational studies), marked associations of health benefits with rises in GWAS-estimated D status often emerge. For example, MR analyses using stratification of cohort data by baseline vitamin D status has already revealed reductions in CVD risks and in mortality rates with GWAS-estimated increases in vitamin D status in subjects with initial 25(OH)D values below ~20 ng/mL. Furthermore, the strongest inverse correlations between health risk and increases in D status are being seen in those with the lowest baseline concentrations of 25(OH)D [37].

The goal of this article is to estimate the potential for reducing disease incidence and death rates in KSA and the United Arab Emirates (UAE) through raising serum 25(OH)D concentrations to above 30 ng/mL (75 nmol/L) for virtually all inhabitants. These two countries were chosen as representative of the Middle East in terms of low mean serum 25(OH)D concentrations and because they provide many studies of the health effects of vitamin D status. Raising minimum 25(OH)D concentrations in these two countries could be achieved through a combination of vitamin D fortification, supplementation and increased, but cautious, solar UVB exposure when the solar elevation angle is greater than 45° [38].

## 2. Approach

This is a narrative review aiming to summarize the evidence on the association of vitamin D deficiency with adverse clinical outcomes for a variety of diseases. Each disease is summarized in a section of this article that includes the best available evidence on the potential health benefits of raising vitamin D status with reference to the respective diseases in the Middle East and elsewhere, up to February 2023. Studies performed in KSA and the UAE are considered, when available and relevant to this study.

The approach taken in this review is first to assess the prevalence of each disease of interest in KSA and the UAE, to identify some of the important risk factors for those diseases and then to discuss the evidence that vitamin D reduces risk of the relevant adverse health outcomes in a causal manner, before identifying those studies judged to provide 25(OH)D concentration-health outcome data suitable for evaluating the expected benefits of raising serum 25(OH)D concentrations to above 30 ng/mL in KSA and the UAE. Inclusion criteria for articles supporting the use of vitamin D in this review required the use of appropriate methodologies and the identification of beneficial effects of vitamin D. As most RCTs have been based on guidelines for pharmaceutical drugs rather than nutrients [32,33], they were largely excluded. Therefore, most of the studies included in this review are observational studies, but MR studies are also included. More recent publications and meta-analyses were generally prioritized. When possible, papers available without cost were selected. These relationships can then be used, together with what is known about the frequency distribution of 25(OH)D concentrations in both countries, for adults and, if available, for the elderly populations of those countries. Since most of the known 25(OH)D concentration-health outcome relationships come from prospective studies with follow-up times between five to ten years, the relationship data may underestimate the benefits of vitamin D due to variation in serum 25(OH)D concentrations with time, as has been shown for all-cause mortality rate [39] and for cancer [40]. Thus, the follow-up time will be considered and the findings from prospective studies adjusted if a clear trend towards a greater effect of serum 25(OH)D concentration is found for shorter follow-up times. Moreover, case-control (CC) results are used when relevant in the absence of other data.

## 3. Disease Mortality Rates in KSA and the UAE

To guide the preparation of this article, mortality rate data for 2016 from KSA and the UAE were obtained from the World Health Organization [41]. Data for the leading causes of death are given in Table 1 for males and females for the two countries. The potential roles of vitamin D in reducing risk of incidence and mortality rates for the various diseases listed as well as all-cause mortality rate are emphasized in this article.

## 4. Metabolic Syndrome

The constellation of abnormalities used to identify the metabolic syndrome [MetS], is the presence of three or more of the following disorders: centrally distributed obesity, dyslipidemia (decreased high-density lipoprotein cholesterol (HDL-C), elevated LDL-C and triglycerides), elevated blood pressure (BP), and hyperglycaemia [42]. Associated with a three-fold and two-fold increased risk of type 2 diabetes and cardiovascular disease (CVD), respectively, this syndrome is thought to be a major driver of the currently increasing epidemics of diabetes and of CVD, and has become a major public health challenge globally. [43].

A study of the MetS and cardiometabolic risk factors was conducted in KSA that included 648 participants from the “general population” who visited the Specialized Polyclinic of Abha, Asir, KSA [44]. The patients had high rates of the features of MetS: 69.4% diabetes and 92.2% obesity. The rate of arterial hypertension was 33% for men and 23% for women, respectively. The prevalence of obesity in KSA is estimated at 41% for men and 49% for women [45], and those participants were apparently less healthy than the total population of KSA; in that group, 60% of the men and 65% of the females had type 2 diabetes mellitus (T2DM) based on an HbA1c > 6.4%. The rate of 25(OH)D < 20 ng/mL was 54% for men and 55% for women. The rate of low HDL-C (<40 mg/dL) was 49% for men and 27% for women, while the rate of high LDL-C (>129 mg/dL) was 29% for men and 21% for women. A study from KSA reported in 2001 found that approximately half of the participants suffered from the MetS [46].

Modest but significant improvements in MetS and its components secondary to a 12-month vitamin D status correction by natural means (sun exposure and vitamin D-rich diet) was observed among 59 non-DM Saudis with vitamin D deficiency [47]. It is noteworthy that in the afore-mentioned study the cohort’s baseline 25(OH)D was only 19.1 ± 1.5 nmol/L which, while increasing to 28.4 ± 1.5 nmol/L after 12 months, still lay within the deficiency range. While the failure to achieve full correction of 25(OH)D status was multifactorial, one major factor unique to the Arab population could be the prevalence of certain VDR polymorphisms, since carriers of *Taq-I* GG and *Bsm-I* TT polymorphisms respond best to vitamin D therapy [48], as do homozygous carriers of the major VDR polymorphisms rs4588 and rs7041) [49].

A review published in 2018 stated that since MetS is about three times more common than diabetes, its global prevalence can be estimated to be about one quarter of the world population [50]. A cross-sectional study of the prevalence of MetS was conducted in the UAE in 2013 [51] among 3212 subjects (mean age 39 ± 11. years old, 74% men, 26% women) who were found to have an overall prevalence of MetS of 39% in men and 37% in women.

By 2012, the evidence regarding vitamin D and MetS [52], suggested that hypovitaminosis D was a risk factor for MetS and its sequelae, T2DM and CVD. In 1998, it had already been suggested that low vitamin D status might contribute to the disorders of MetS, called syndrome “X” at that time [53], since there was already mechanistic and observational evidence suggesting protective effects of vitamin D for MetS though RCT evidence has been weakened by inadequate power, low vitamin D dosages, and supplementing too late in life. Since then, vitamin D supplementation has been shown to reduce cardiac disorders and all-cause mortality as well as cancer mortality [54]. Further bidirectional MR analysis has shown that vitamin D status is reduced by obesity but does not determine obesity though better status may reduce central adiposity [55]. Vitamin D reduces inflammation, a major factor worsening vascular disease as well as increasing tissue damage in infection. Vitamin D also reduces abnormally increased insulin resistance while higher status is associated casually with reduced blood pressure as documented by MR analyses [56]; it also reduces circulating triglycerides and may increase HDL cholesterol [57]. The subsequent sections will provide further discussion on these findings, demonstrating the consistent reporting of inverse associations between plasma or serum 25(OH)D concentrations and the defining characteristics of MetS, including hyperglycemia, dyslipidemia, and high body mass index [58].

In conclusion, while the primary cause of MetS in the Middle East and elsewhere is thought to be related to diet, it may also be worsened by reduced serum 25(OH)D concentrations. Thus, it is tempting to hypothesize that vitamin D supplementation should improve the features of MetS or reduce their risks over time in populations where vitamin D deficiency is prevalent [59]. This is important as MetS is a major risk factor for both T2DM and CVD.

## 5. Arterial Hypertension

In 2010 it was estimated that the prevalence of arterial hypertension among adults globally was 31% and it is one of the major causes of mortality [60]. A study conducted in KSA in 2013 measured systolic and diastolic blood pressure of 10,735 Saudis aged 15 years or older [61] and found that 15% and 41% of subjects were hypertensive or borderline hypertensive, respectively, and that 58% of hypertensive Saudis were undiagnosed. Other reports showed hypertension at 22% amongst women based on a review of articles from 2000 to 2015 [62]; 31% among KSA nationals vs. 55% among expatriates in a study of 550 participants whose blood pressure was measured in 2011 and 2012 [63]. Reported arterial hypertension rates in the UAE were 31% (95% CI, 27–36%) between 2005 and 2021 with 37% (27–36%) in Abu Dhabi and 29% (24–35%) in Dubai and 30% among 6193 individuals from the UAE, Northern Africa and Middle East with a mean age of 39 ± 13 years screened in May 2017 [64].

One of the reasons hypertension rates should be reduced is that it is associated with increased risk of death from several diseases including, in order of death rates globally, ischemic heart disease, hemorrhagic stroke, other CVD events, ischemic stroke and chronic kidney disease [60].

Risk factors for hypertension include age, obesity, type 2 diabetes, and hypercholesterolemia, [61], obesity being especially important [65]. Fresh fruits and vegetables lower the risk of hypertension while high sodium intakes raise risk [66]. Ultra-processed foods, which have a high energy density and are rich in salt, sugar and fat, are also an important risk factor for hypertension based on a review of eight observational studies with ultra-processed foods comprising 8% to 56% of the energy content of individual diets [67].

An observational study from Harvard examined the risk of incident hypertension with respect to serum 25(OH)D concentrations [68]. Two prospective cohort studies including 613 men from the Health Professionals’ Follow-Up Study and 1198 women from the Nurses’ Health Study with measured 25(OH)D concentrations followed for 4 to 8 years. During 8 years of follow-up, the multivariable RR of incident hypertension among men whose measured plasma 25(OH)D levels were <15 ng/mL compared with those whose levels were ≥30 ng/mL was 3.53 (95% CI: 1.02 to 12.3). Amongst women, the same comparison showed a RR of 1.70 (95% CI: 0.92 to 3.16). While that study suggested that higher 25(OH)D concentrations are associated with reduced risk of arterial hypertension, it was not designed to distinguish between the effects of vitamin D and those of nitric oxide, both of which are increased through UVB exposure.

In 2009, a meta-analysis of 11 RCTs was conducted on vitamin D supplementation and change of blood pressure [69]. The meta-analysis of the four studies with hypetensives supplemented with 800 to 2900 IU/d vitamin D_3_ or UVB radiation resulted in a decrease in systolic blood pressure of −6.2 mmHg, 95% CI −12.3 to −0.04 mmHg) and a similar reduction was seen in diastolic blood pressure (−3.1 mmHg, 95% CI −5.5 to −0.6 mmHg).

An interventional observational study conducted in Canada found that raising serum 25(OH)D concentration above 40 ng/mL (on 4000–5000 IU/day of vitamin D_3_) lowered blood pressure and reduced the prevalence of hypertension among those with hypertension but did not affect blood pressure in normotensives [70]; significant increases in mean serum 25(OH)D (35 ± 15 to 45 ± 16 ng/mL) were found whilst mean blood pressure variables remained unchanged [systolic BP 125 ± 17 mmHg to 125 ± 17 mmHg (*p* = 0.10); diastolic BP 77 ± 10 mmHg to 77 ± 9 mmHg (*p* = 0.10)]. Of those that were hypertensive at baseline (*n* = 592), 71.1% (*n* = 421) were no longer in the hypertensive range at follow-up. Amongst those with hypertension, 44% and 49% were on BP-lowering medication at baseline and at follow-up, respectively. There was no significant difference in the reduction of systolic blood pressure (−12.7 ± 20.6 mmHg vs. −13 ± 18, *p* = 0.7) and diastolic blood pressure (−11 ± 11 mmHg vs. −10 ± 10, *p* = 0.07) between participants who did and did not use BP-lowering medication. However, >9% of participants who were hypertensive and on BP-lowering medication at program entry were able to discontinue hypotensive medication by the end of their year in the program.

Two subsequent clinical trials conducted in Brazil also found that high-dose vitamin D_3_ supplementation significantly reduced blood pressure. One involved 43 T2DM patients [71]. Half of the participants were given a single dose of 100,000 IU vitamin D_3_ and half were given a placebo. Serum 25(OH)D increased from 14 ± 5 ng/mL to 23 ± 7 ng/mL, *p* = 0.02 at eight weeks in the treated group and from 15 ± 5 ng/mL to 19 ± 5 ng/mL, *p* = 0.6 in the control group. At eight weeks, daytime SBP was lower in the treated than in the placebo group (−7 vs. −1, *p* = 0.007), as was daytime DBP (−5 vs. 0. *p* = 0.01). The second trial involved 35 T1DM normotensive patients of mean age 26 ± 11 years with mean baseline 25(OH)D concentration = 26 ± 8 ng/mL. Depending on baseline 25(OH)D concentration, they were given 4000 or 10,000 IU/d vitamin D_3_ for three months to achieve 25(OH)D concentration between 30 and 100 ng/mL. The mean achieved 25(OH)D concentrations was 52 ± 25 ng/mL. Significant reductions were found only for morning SBP (117 ± 14 mmHg reduced to 112 ± 14 mmHg, *p* < 0.05) and DBP (74 ± 9 mmHg reduced to 70 ± 10 mmHg, *p* < 0.05).

A retrospective chart review was made to evaluate 5308 naïve [previously untreated] hypertensive adults registered for treatment across Abu Dhabi Health Services (SEHA) clinics in Abu Dhabi in 2017 [72]. After collecting data for baseline details and BP measurements, patients were followed up for six months. Patients who did not reach BP targets despite taking three or more antihypertensive medications were defined as treatment-resistant hypertensive. The overall adherence to antihypertensive treatment was 42%. At 6 months, a significant reduction in BP was observed in patients adherent to their medications (SBP: −4.5 mmHg and DBP: −5.9 mmHg). Among 189 patients, using three or more antihypertensive medications for six months, only 34% were adherent to their treatment, and only 14% reached the BP target. The prevalence of treatment-resistant hypertension was 20%. Thus, it appears that blood pressure-lowering drugs do not reliably reduce the prevalence or severity of hypertension in the UAE. These data, however, suggest that correcting vitamin D deficiency could provide a useful adjunctive measure for reducing BP in the UAE, in addition to managing other secondary causes of arterial hypertension.

## 6. Cardiovascular Disease

The global prevalence of identified CVD disease was 523 million (95% UI: 497 to 550 million) in 2019, when the number of recorded CVD deaths/year reached 18.6 million (95% UI: 17.1 to 19.7 million). CVD remains the leading contributor to disease burden globally. There is an urgent need to focus on implementing existing cost-effective policies and interventions if the world is to meet the targets for Sustainable Development Goal 3 and achieve a 30% reduction in premature mortality from noncommunicable diseases [73].

The role of vitamin D in reducing cardiovascular disease (CVD) risks was first hypothesized by Robert Scragg in 1981 based on the higher rate of CVD deaths in winter than in summer [11]. He later reported that serum 25(OH)D concentration was inversely correlated with risk of MI in a CC study [12]. A study reported in 1991 that seasonal variations of all-cause mortality rate were similar in north-east Scotland and Kuwait [74], the IHD mortality rates during 1981–1984 and 1986–1988 being ~35% higher in winter than in summer in Kuwait and ~30% higher in Scotland in winter than summer during 1974–1988. It was noted that temperature varies in Kuwait from 45 °C in summer to 20 °C in winter and from 18 °C in summer and to 5 °C in winter in Scotland. The authors had no explanation for their findings. However, a report in 2022 showed that seasonal changes in serum 25(OH)D concentrations had the highest impact on seasonal variations in mortality rates, with seasonal changes in temperature having a similar but lesser effect [75].

It took an observational study based on data from the Framingham Offspring Study reported in 2008 to make researchers aware of the potential role of vitamin D in reducing the risk of CVD [16]. That study included 1739 participants of whom 120 developed a first CVD event during a 4.5-year follow-up period. Individuals with 25(OH) D < 15 ng/mL had a multivariable-adjusted hazard ratio (aHR) = 1.62 (95% CI, 1.11 to 2.36, *p* = 0.01) for incident CVD events compared with those with 25(OH)D ≥ 15 ng/mL. Since then, many observational studies have reported similar findings. By 2012, 19 independent studies with 6123 CVD cases in 65,994 participants were included in a meta-analysis [76]. Comparing those in the lowest vs the highest 25(OH)D categories, the pooled RR was 1.52 (95% CI, 1.30–1.77) for total CVD; 1.42 (95% CI, 1.19–1.71) for CVD mortality; 1.38 (95% confidence interval, 1.21–1.57) for coronary heart disease, and 1.64 (95% CI, 1.27–2.10) for stroke. Another meta-analysis published in 2019 [77] examined 25 studies with 10,099 cases of CVDs, showing increased risks of CVD mortality [relative risk (RR)  =  1.54, 95% CI: 1.29–1.84)] and incidence rates (RR  =  1.18, 95% CI: 1.00–1.39) with lower vitamin D status.

An observational study from northern European countries included 26,916 participants (median age 62 years, 58% females) with a median 25(OH)D concentration of 54 nmol/L [78]. During a median follow-up time of 10.5 years, 6802 subjects died. Compared to participants with 25(OH)D concentrations of 30 to 40 ng/mL, the aHRs for mortality in the groups with 25(OH)D values of 16 to 20, 12 to 16, and <12 ng/mL were 1.15 (95% CI, 1.00–1.29), 1.33 (95% CI, 1.16–1.51), and 1.67 (95% CI, 1.44–1.89), respectively, with similar increases in CVD mortality with lower serum 25(OH)D concentrations.

A study based on the UK Biobank data included 365,530 participants who had serum 25(OH)D measurements and no history of CVD, cancer, or diabetes at baseline (2006–2010) [79]. During a median follow up of 8.9 (interquartile range (IQR): 8.3–9.5) years, 10,175 deaths occurred, including 1841 due to CVD and 5737 (56.4%) due to cancer. The multivariate analyses revealed nonlinear inverse associations, with a decrease in mortality risk appearing to level off at or above 24 ng/mL of 25(OH)D for all-cause and CVD deaths and at or above 18 ng/mL for cancer deaths. Compared to participants with 25(OH)D concentrations below those cutoffs, those with higher concentrations had a 17% lower risk for all-cause mortality (hazard ratio [HR]: 0.83, 95% CI: 0.79–0.86), a 23% lower risk for CVD mortality (HR: 0.77, 95% CI: 0.68–0.86), and an 11% lower risk for cancer mortality (HR: 0.89, 95% CI: 0.84–0.95).

A retrospective, observational, nested CC study based on participants who received care at the U.S. Veterans Health Administration examined the effect of vitamin D supplementation in reducing myocardial infarction (MI) events between 1999 and 2018 [80]. Participants in different 25(OH)D categories were matched using propensity score. It was found that those prescribed vitamin D supplements and with achieved serum 25(OH)D concentration > 30 ng/mL had a significantly reduced risk of MI events than those who were not supplemented and remained <20 ng/mL [HR 0.73 (95% CI 0.55–0.96)] or between 20 and 30 ng/mL [HR = 0.65 (95% CI 0.49–0.85)].

RCTs have not supported a role for vitamin D supplementation in reducing risk of CVD [81]. None of the 21 RCTs discussed in this meta-analysis reported a significant reduction of CVD from vitamin D supplementation. The primary reason seems to be that participants in such trials have had median 25(OH)D concentrations around 30 ng/mL, which is much higher than concentrations at which significant risks of CVD have been found (see above).

A recent vitamin D supplementation trial conducted in KSA involving 120 participants of whom 32 had 25(OH)D concentrations <10 ng/mL and 88 had 25(OH)Ds between 10 and 20 ng/mL [82]. Those in the treatment arm were given 50,000 IU/week vitamin D_3_ for two months, then twice a month, followed by daily 1000 IU until month 6, while those in the control arm were given a placebo. The goal was to see whether this approach reduced their 10-year risk of Atherosclerotic Cardiovascular Disease (ASCVD) risk scores. Only 25 participants achieved >30 ng/mL. Only HDL cholesterol showed favorable significant changes in the participants, which translated to significantly improved 10-year ASCVD risk scores: for achieved 25(OH)D concentration < 30 ng/mL, −32%, *p* = 0.004; for achieved 25(OH)D >30 ng/mL, −47%, *p* = 0.002.

However, strong evidence that vitamin D reduces risk of CVD in a causal manner in the long term comes from a recent MR study [83]. It used a non-linear MR analysis of UK Biobank data for 44,519 CVD cases and 251,269 controls. Genetically determined increases in serum 25(OH)D concentration were instrumented using 35 GWAS-confirmed significant gene variants for subjects with baseline serum 25(OH)D values stratified into 100 ranges. There was a striking L-shaped association between genetically predicted increases in serum 25(OH)D and CVD risk (*p*, non-linear = 0.007), reductions in CVD risk being seen with a genetically determined increase in serum 25(OH)D in subjects with baseline 25(OH)D values below <10 ng/mL with the OR for a CVD event being >2.0 for those with the lowest baseline 25(OH)D values, falling to near 1.0 near 10 ng/mL. Examination of those data shows that raising serum 25(OH)D for those with deficiency to ≥10 ng/mL could reduce CVD risk by 2%, and raising it to ≥20 ng/mL could reduce CVD risk by 4%, though raising 25(OH)D to >40 ng/mL was unlikely to increase this benefit further. However, MR analyses probably underestimate predictions of the benefits of raising serum 25(OH)D concentration since they use genetically-determined 25(OH)D estimates based on only a few of the genes involved in the vitamin D pathway. However, since the MR analysis approach randomizes participants based on predicted 25(OH)D concentrations, it is becoming accepted as being as useful as RCTs for determining causality [37].

In 2016, the results of an observational study on the incidence of stroke with respect to serum 25(OH)D concentrations were reported in the Reasons for Geographic and Racial Differences in Stroke (REGARDS) study, which included both black and white adults [84]. Participants were enrolled between 2003 and 2007 and were followed until September 2011. The study found that individuals with a baseline 25(OH)D concentration <20 ng/mL had an adjusted hazard ratio (aHR) of 1.85 (95% CI, 1.17–2.93) for stroke compared to those with a concentration >30 ng/mL. For those with a concentration of 20–30 ng/mL, the aHR was 1.33 (95% CI, 0.89–1.96). Similar results were observed for both ischemic and hemorrhagic stroke.

The prospective study from the U.S. veterans [80] forms the basis for the estimate of reduction in risk of MI incidence from vitamin D supplementation to achieve 25(OH)D >30 ng/mL applied to the UAE using the serum 25(OH)D concentration data of Haq et al. [85]. The result, given in Table 2, indicates that risk of MI in the UAE could be reduced by 25% (95% CI, 10–40%).

For the KSA, the data used are from a cross-sectional series of measurements of 25(OH)D concentrations from about 500 females and 400 males with mean age rising from 26 ± 16 years in 2008 to 36± each year from 2008 to 2017 [86]. The trends showed that prevalence of 25(OH)D concentration for vitamin D deficiency [25(OH)D] between 12 and 20 ng/mL decreased from 87% in 2008 to 59% in 2017, while the prevalence of severe vitamin D deficiency (25(OH)D concentrations <12 ng/mL) decreased from 48% in 2008 to 17% in 2017. The values in 2017 were used in the calculations for the KSA (Table 3). The calculations could easily be revised when more recent data for older inhabitants of KSA become available.

The prospective observational study of incident stroke in the U.S. [84] forms the basis for the estimate of stroke reduction in the UAE by raising the minimum serum 25(OH)D concentration to >30 ng/mL. The result for the UAE, given in Table 4, is that risk of stroke could be reduced by 35% (95% CI, 7–53%). The result for KSA, given in Table 5, is that risk of stroke could be reduced by 40% (95% CI, 9–60%).

Two studies reporting mortality rates for CVD with respect to serum 25(OH)D concentrations were used: one based on data from northern Europe [78], the other on results from the UK [79]. The result for UAE, given in Table 6, is that risk of CVD mortality could be reduced by 32% (95% CI, 19–40%). The result for KSA, given in Table 7, is that risk of CVD mortality could be reduced by 37% (95% CI, 15–47%).

The result for UAE, given in Table 8, is that risk of CVD mortality could be reduced by 17% (95% CI, 8–25%). The result for KSA, given in Table 9, is that risk of CVD mortality could be reduced by 24% (95% CI, 24–33%). The difference in the two estimates for reduction in CVD mortality rate indicates that the approach used for the calculations depends strongly on the data used.

## 7. Diabetes Mellitus

The global prevalence of T2DM was estimated at 462 million in 2017 with one million deaths/year attributed to T2DM [87]. The rate of T2DM rises from 4.4% for those aged 14–49 to 22% for those over 70 years. Diabetes rates in KSA and the UAE estimated by the International Diabetes Association for 2021 are: for KSA, 4.3 million with diabetes (19% age-age adjusted) and 1.9 million with undiagnosed diabetes, 3800 with T1DM (https://diabetesatlas.org/data/en/country/208/ae.html, accessed on 1 February 2023); for UAE, 1.0 million with diabetes (16% age-age adjusted) and 0.6 million with undiagnosed diabetes, 400 with T1DM (https://www.diabetesatlas.org/data/en/country/174/sa.html, accessed on 1 February 2023).

Conditions frequently reported with T2DM in the UAE study include hypertension (83%), obesity (59%), retinopathy (17%), and microvascular complications (20%). Kidney damage was found in 52% of the patients, and kidney failure in 2% [88].

Increased oxidative stress appears to be a deleterious factor causing insulin resistance, dyslipidemia, β-cell dysfunction, and impaired glucose tolerance and ultimately leads to T2DM [89]. Oxidative stress is the excessive production of various oxidant species and has an important role in aggravating inflammation and in the pathophysiology of a number of debilitating illnesses, including cardiovascular diseases, diabetes, cancer, and many neurodegenerative processes [90].

T2DM is a disease in which the development of increasing insulin resistance leads to higher circulating insulin levels, needed to overcome insulin resistance in the tissues. Over time this leads to islet beta cell failure with reducing ability to produce enough insulin to assure glucose homeostasis, followed by fasting hyperglycemia and progressing to glucose intolerance and then to type 2 diabetes. Increased insulin resistance is a major factor in the pathogenesis of type 2 diabetes and plays a key role in the development of associated metabolic abnormalities such as dyslipidemia and dysglycemia and also increases the risks of CVD [91].

That the combination of increased insulin resistance with worsening β-cell function over time leads to worsening glucose tolerance and then to type 2 diabetes has been confirmed many times since first postulated as the problem underlying the development of T2DM by Harold Himsworth [92]. The negative effects of obesity are partly due to the release of adipokines, which can worsen inflammation. Mechanistic research provides evidence that vitamin D may have the potential to alleviate tissue damage resulting from inflammation caused by obesity [93].

Several prospective studies have found that vitamin D status affects the risks of developing T2DM. A 12-year follow-up observational study was conducted in southern California on 903 persons known to be free of diabetes or prediabetes in 1997–1999. During follow-up, 47 cases of T2DM and 337 cases of prediabetes were detected. The HR for progression to T2DM for a 25(OH)D > 50 ng/mL vs. <30 ng/mL was 0.19 (95% CI, 0.06–0.56), and per 10 ng/mL increase in 25(OH)D it was 0.64 (95% CI, 0.48–0.86) [94]. A study in Ireland involving 5272 adults over the age of 50 years followed for four years found an increased likelihood [RRR = 1.62 (95% CI, 1.12–2.35)] of progressing to prediabetes from normoglycemia for 25(OH)D < 12 ng/mL compared to >30 ng/mL [95].

A secondary analysis of results from the D2d RCT for conversion from prediabetes to T2DM [35] on participants in the treatment arm who were given 4000 IU/d vitamin D_3_ is of relevance. The results of the trial in terms of intention to treat, were not significant [34] but, in analyses based on achieved 25(OH)D concentration amongst subjects in the treatment arm, the risk of developing T2DM in those who achieved and maintained intra-trial 25(OH)D levels of 40–50 ng/mL or >50 ng/mL were 0.48 (0.29–0.80) and 0.29 (0.17–0.50), respectively, vs. T2DM risk in those maintaining levels of 20–30 ng/mL.

Since we cannot make final conclusions on causality based on observational studies, and RCTs have largely failed, it is useful to use MR studies which are considered as equally reliable for evaluating causality. An MR study published in 2018 used data of four 25(OH)D single nucleotide polymorphisms (SNPs; *n* = 82,464), plasma 25(OH)D concentrations (*n* = 13,565), and cases with diabetes (*n* = 5565) in the China Kadoorie Biobank (CKB) [96]. The effects on risk of diabetes were assessed by a genetic score using two 25(OH)D synthesis SNPs (DHCR7-rs12785878 and CYP2R1-rs10741657), with and without the addition of SNPs affecting the transport (GC/DBP-rs2282679) and catabolism (CYP24A1-rs6013897) of 25(OH)D. The CKB results were combined in a meta-analysis of data from 10 studies for the two synthesis SNPs (*n* = 58,312 cases) and 7 studies for all four SNPs (*n* = 32,796 cases). Mean (SD) 25(OH)D concentration was 65 (8) ng/mL in CKB, and the per allele effects of genetic scores on 25(OH)D were +2.87 (SE 0.39) ng/mL for the synthesis SNPs and +3.54 (SE 0.32) ng/mL for an analysis using four SNPs. A 10-ng/mL higher biochemically measured plasma 25(OH)D concentration was associated with a 9% (95% CI: 0–18%) lower risk of diabetes in CKB. In a meta-analysis of all studies, a 10-ng/mL higher genetically instrumented 25(OH)D concentration was associated with a 14% (95% CI: 3–23%) lower risk of diabetes using the 2 synthesis SNPs. In view of the increasing global epidemic of T2DM such significant reductions are of practical relevance for public health [97].

A review published in 2019 reported on vitamin D deficiency among T2DM patients in KSA [98]. A total of 12 Saudi articles with a total of 14,645 patients were included. The majority of those articles reported that the prevalence of vitamin D deficiency was high among T2DM patients, particularly among older patients, women of childbearing age, and younger males, the prevalence ranging from 38% to 80%.

Given that vitamin D deficiency is associated with increased risk of developing T2DM, it seems reasonable to expect that vitamin D supplementation in patients with prediabetes could increase the natural reversion rate of prediabetes to normoglycemia. The earlier demonstration that supplementation with 4000 IU/d vitamin D_3_ for six months in vitamin D deficient but normoglycemic south Asian women to 25(OH)D values of 32 ng/mL or above reduced insulin resistance to normal supports this view [99], as do the reduced T2DM rates seen with high achieved 25(O)D levels in the D2d study [35]. Furthermore, this benefit was found in non-obese subjects (RR 0.73 [95%CI 0.57–0.92]) but not in obese subjects (RR 0.95 [95% CI 0.84–1.08]) (*p*, interaction = 0.048). This lack of an effect on obesity could well reflect the reduced hepatic 25-hydroxylaton of vitamin D and the increased secretion of FGF-23 secretion seen in obesity—the latter reducing activity of the vitamin D-activating 1-alpha hydroxylase and thus reducing calcitriol formation [100]. Reversion of prediabetes to normoglycemia was found in 116 of 548 (21.2%) participants in the vitamin D group and 75 of 532 (14.1%) in the control group; thus, supplementation increased the reversion rate of prediabetes to normoglycemia [RR 1.48 (95% CI 1.14–1.92)]. In addition, diabetes also reduces hepatic 25-hydroxylase activity [100]. Thus, the provision of extra vitamin D may be as important as the correction of hyperglycemia in the treatment of diabetes.

In 2022 it was reported that high-dose vitamin D_3_ (HDVD) supplementation (50,000 IU/week) for three months reduced insulin resistance in Saudi females while low-dose vitamin D_3_ (LDVD) (25,000 IU/week) did not [101]. Most anthropometric variables varied non-significantly between the two groups. For example, mean BMI in the LDVD arm was 28.6 ± 1.5 kg/m^2^, while that in the HDVD arm was 26.8 ± 1.5 kg/m^2^, (not significant). Serum 25(OH)D concentrations increased non-significantly from 16 ± 2 ng/mL to 23 ± 3 ng/mL in the LDVD group and significantly from 13 ± 1 ng/mL to 40 ± 3 ng/mL in the HDVD group. Importantly, fasting serum insulin levels and Homeostatic Model Assessment for Insulin Resistance (HOMA-IR) did not change significantly in the LDVD group but decreased significantly by 13 ± 8 µIU/mL in the HDVD group as did HOMA-IR, from 2.9 ± 0.2 to 1.8 ± 0.1. The statistical analysis found that HOMA-IR was non-significantly reduced in the LDVD arm (−0.13) and −0.31 (*p* < 0.05) in the HDVD arm.

In view of the data discussed above, it is surprising that neither ‘A Consensus Report from the American Diabetes Association’ (ADA) nor the ‘European Association for the Study of Diabetes (EASD)’ report [102] make any mention of vitamin D in relation to T2DM.

An adjuvant study of vitamin D supplementation on T2DM patients was conducted in KSA [103]. A total of 34 men (57 ± 9 years, BMI = 29 ± 3 kg/m^2^) and 58 women (51 ± 11 years, BMI = 34 ± 5 kg/m^2^) were given 2000 IU/d vitamin D for 18 months. Serum 25(OH)D concentrations rose from baseline (13 ± 1 ng/mL) to 18 months (22 ± 1 ng/mL). A significant decrease in LDL- (baseline = 4.4 ± 0.8 mmol/L vs. 18 months = 3.6 ± 0.8 mmol/L, *p* < 0.001) and in total cholesterol (baseline = 5.4 ± 0.2 mmol/L vs. 18 months = 4.9 ± 0.3 mmol/L, *p* < 0.001) were noted, as well as a significant improvement in HOMA-β function (*p* = 0.002). Most of these benefits were more prominent in women than men.

A cross-sectional study in Jazan City, KSA, studied vitamin D status and glycemic control among 309 T2DM patients [104]. The mean age was 59 ± 12 years and the mean 25(OH)D concentration was 19 ± 7 ng/mL. Of the 130 males, 49% were vitamin D deficient, and 69% of the 179 females were vitamin D deficient. Figure 2 in [104] is a plot of HbA1C vs. 25(OH)D concentration where the slope of the linear fit to the data is ~−0.11 HbA1C/[25(OH)D] and the regression coefficient, *r* = 0.44, *p* < 0.001.

## 8. Chronic Kidney Disease

A number of important risk factors have been identified for chronic kidney disease (CKD), including African-American descent, male gender, older age, and family history [105]. Other major risk factors include smoking, obesity, hypertension, and diabetes mellitus. Obesity raises blood pressure by increasing tubular sodium reabsorption, altering renal compression [106] and hence reducing natriuresis which causes volume expansion via activation of the sympathetic nervous system and the renin-angiotensin-aldosterone system, and increased physical compression of the kidneys, especially with increased visceral adiposity [107]. In a recent study based on the UK Biobank dataset, new onset CKD was found to be inversely correlated with 25(OH)D concentrations for participants with diabetes [HR = 0.91 (95% CI, 0.86–0.96) per standard deviation (approximately 10 ng/mL)], but not for non-diabetic participants [108].

### 8.1. Renal Outcomes Due to Obesity

As mentioned above, obesity raises blood pressure by various mechanisms. Other factors such as inflammation, oxidative stress, and lipotoxicity may also contribute to renal dysfunction with obesity-mediated hypertension. Initially though, obesity causes renal vasodilation and glomerular hyperfiltration, which act as compensatory mechanisms to maintain sodium balance despite increased tubular reabsorption. However, these compensations, along with increased arterial pressure and metabolic abnormalities, may ultimately lead to glomerular damage, initiating a slowly progressive vicious cycle that exacerbates hypertension and eventually reduces renal function [107].

### 8.2. Renal Outcomes with Low 25(OH)D in CKD

A study conducted in Canada tested the rationale for raising target serum 25(OH)D concentration guidelines for clinical practice in the management of CKD [109]. Participants were in Stage 3 or 4 CKD and had BMIs averaging 34–35 kg/m^2^. They were treated with slow-release calcifediol [i.e., 25(OH)D)] or placebo in 26-week prospective RCTs. Suppression of PTH and bone turnover markers occurred when 25(OH)D reached between 51 ng/mL and 93 ng/mL [109]. It is not known what the optimal PTH is for CKD. As noted in that article, a PTH of 65 pg/mL is the upper limit of normal, and a recent commentary reported that a PTH > 65 pg/mL is associated with increased risks of heart failure and CVD death, but not an all-cause mortality rate [110]. However, a meta-analysis reported in 2016 that elevated PTH was a significant risk factor for all-cause mortality rate but, again, not for CVD mortality rate [111].

A meta-analysis of ten studies found mortality rates associated with CKD with respect to a 10 ng/mL increase in 25(OH)D had a RR = 0.86 (95% CI, 0.82–0.91) [112]. Among those ten studies were two that can be used to estimate mortality rate as a function of 25(OH)D concentration. One, from the U.S, used a cohort of 30,122 patients from the Third National Health and Nutrition Examination Study who had CKD but were not on dialysis and who had a mean follow up of nine years [113]. At baseline, mean BMI was ~27 ± 5 kg/m^2^ and mean age ~55 years; the percentages of participants in each CKD stage were 44% in Stage 1, 27% in Stage 2, 27% in Stage 3, and 1% in Stage 4. The aHRs for mortality rate by 25(OH)D concentrations were, versus 25(OH)Ds > 30 ng/mL, 1.17 (95% CI, 0.99–1.38), for 25(OH)Ds of 15–30 ng/mL and 1.56 (1.23–2.18) if 25(OH)Ds were <15 ng/mL. The other study was based on 1108 diabetic hemodialysis patients participating in the German Diabetes and Dialysis Study [114]. At baseline, mean BMI was ~27 ± 5 kg/m^2^ and the mean duration of diabetes was between 16 and 20 years (SD, 7 years). For those with 25(OH)D ≤10 ng/mL vs. >30 ng/mL, the aHR for all-cause mortality rate was 1.65 (95% CI, 1.14–2.38); for those with 25(OH)Ds between 10 and 20 ng/mL, the aHR was 1.20 (95% CI, 0.86–1.68), and for those between 20 and 30 ng/mL, the aHR was 1.16 (95% CI, 0.80–1.70).

### 8.3. Role of Vitamin D Treatment in CKD

Since the kidney is an organ that converts 25(OH)D to circulating calcitriol, whose concentrations are important in regulating serum calcium concentrations and for bone health [115], untreated CKD results in increases in PTH levels. A meta-analysis of ten studies using oral calcitriol [natural or analogue] treatment of hemodialysis patients found significant reductions in all-cause mortality, aRR = 0.73 (95% CI, 0.64–0.83) while for CVD mortality from four studies, aRR = 0.55 (95% CI, 0.41–0.74) [116].

A systematic review and meta-analysis of responses to vitamin D supplementation for patients with CKD was published in 2021 [117]. It examined five studies giving vitamin D_2_ or D_3_, finding a non-significant reduction of PTH [−18 pg/mL (95% CI, −37 to 2 pg/mL)], while the analysis of six trials using calcitriol or calcitriol analogues found a significant reduction of PTH [−35 pg/mL (95% CI, −60 to −10 pg/mL)]. The trial with the best outcome was conducted in Sweden [118]. This trial had 47 participants in the vitamin D arm and 48 in the control arm and ran for 21 weeks; the mean ag was 63 ± 14 years, the mean BMI 29 ± 6 kg/m^2^, and about half were in CKD Stage 3 and half in CKD Stage 4. For those in the vitamin D treatment arm, the mean serum 25(OH)D increased from 23 ± 9 ng/mL to 65 ± 20 ng/mL, and PTH decreased from 10.9 ± 5.0 to 10.5 ± 5.0 pmol/L; in the control arm however, 25(OH)D increased from 23 ± 9 ng/mL to 25 ± 10 ng/mL and PTH increased from 13 ± 9 pmol/L to 15 ± 11 pmol/L. In subgroup analyses, for those in the CKD Stage 3 subgroup PTH did not change in either treatment arm; for those in the CKD Stage 4 subgroup, the mean PTH changed from 13  ±  7 to 12  ±  6 pmol/L in the treatment group, and from 16  ±  11 to 19  ±  12 pmol/L in the placebo group. The mean (confidence interval) difference in the change in mean PTH between groups was −3.8 (−6.5; −1.1) (*p*  =  0.006).

## 9. Alzheimer’s Disease

Evidence that vitamin D reduces Alzheimer’s disease (AD) risks in a causal manner comes from MR studies. The first report that vitamin D was causally linked to AD was published in 2016 [119]. It used data for four single nucleotide polymorphisms (SNPs) from the largest database then available, from a GWAS for vitamin D (the Study of Underlying Genetic Determinants of Vitamin D and Highly Related Traits [SUNLIGHT] Consortium; N 533,996) [120] together with data on 17,008 clinically determined AD patients and 37,154 controls from the International Genomics of Alzheimer’s Project [IGAP] [121]. That MR analysis demonstrated that a 1 standard deviation decrease in natural log-transformed serum 25(OH)D increased AD risk by 25% (odds ratio 1.25, 95% confidence interval 1.03–1.51, *p* = 0.02). (A 1 standard deviation of 25(OH)D was approximately 10 ng/mL).

Another MR study used data from IGAP and the UK Biobank [122] and reported, from the IGAP data, significant inverse correlations between both genetically determined and laboratory measured 25(OH)D concentrations with AD in prospective studies, while neither measure of 25(OH)D concentration was inversely related to AD risk in the UK Biobank dataset. The proposed reason for this discrepancy was that IGAP AD data were based on clinical diagnoses while the UK Biobank AD data were based on self-reported AD diagnoses or by-proxy from family members.

A recent article reported measurements of 25(OH)D in the serum and in the brain with respect to mild cognitive impairment or dementia [123]. This study was conducted among 290 participants in the Descendants of the Rush Memory and Aging Project with a mean age at death of 92 ± 6 years and a mean plasma 25(OH)D concentration of 35 ± 17 ng/mL. At the final cognitive diagnosis, 41% had dementia, 24% had mild cognitive impairment (MCI), and 36% had no MCI. The odds of having dementia or MCI at the last cognitive assessment before death were 25% to 33% lower per doubling of 25(OH)D_3_ in the four brain regions measured (OR 0.67 to 0.75, *p* ≤ 0.03 for all analyses), but plasma 25(OH)D concentrations were not significantly associated with global cognitive function or global cognitive decline; however, mean plasma 25(OH)D concentration was so high at 35 ± 17 ng/mL that this study would be unlikely to have been able to reveal variations in cognitive function with vitamin D status at lower concentrations.

Several mechanisms by which the lack of vitamin D would increase the risks of AD have been identified. Such mechanisms, together with evidence that increasing vitamin D provision reduces the magnitude of those effects are listed below (see Table 10).

Findings from observational studies of the incidence of AD and of vascular dementia up to 2017 were reported in 2019 [135] and plotted (Figures 2 and 3 in a recent review [33]) so as to be able to estimate the circulating 25(OH)D concentration–risk relationship. The assumption was made that each study’s findings reflected the HR for the mean 25(OH)D concentration of that study. The study by Littlejohns [136] was omitted from the graphs since it was an outlier from the relationships seen in the other studies. For dementia, the regression fit to that data had an HR = 0.19 +0.031× [25(OH)D ng/mL; 95% CI, ~−0.2, 0.3 for 12 ng/mL—decreasing to 0.1,0.1 at 28 ng/mL]. For AD, the regression fit to the data showed a HR = 0.51 +0.016× [25(OH)D ng/mL; 95% CI, ~−0.1, 0.2 for 25(OH)D values of 28 ng/mL vs. 12 ng/mL]. Those plots suggested, overall, that the optimal 25(OH)D concentration for brain health must be at or above 30 ng/mL.

According to a recent study, vitamin D supplementation significantly reduces the incidence of dementia, including AD [137]. The study analyzed data from the U.S. National Alzheimer’s Coordinating Center database for the years 2005–2021 (https://naccdata.org/ accessed on 14 March 2023). Participants were considered taking vitamin if they had vitamin D supplementation listed on the NACC A4 medication form at time of enrollment. During the ten-year follow-up period, those who took vitamin D supplements had a lower dementia-free survival time [HR = 0.60 (95% CI, 0.55–0.65)]. Since AD is the most common type of dementia, these findings support the use of vitamin D supplementation to reduce the risk of AD. In secondary analyses, a significant reduction was observed among Black people [HR = 0.59 (95% CI, 0.51–0.67)], while other racial/ethnic groups did not show a significant reduction [HR = 0.94 (95% CI, 0.76–1.15)]. These differences by race/ethnicity are likely due to variations in 25(OH)D concentrations.

## 10. Cancer

A role of vitamin D in reducing cancer risks was proposed in 1980 by the brothers Cedric and Frank Garland, based on their finding that colon cancer mortality rates were inversely correlated with annual solar radiation exposures in the U.S. [7]. They reasoned that since the most important physiological role of solar radiation was vitamin D production, vitamin D provision should play a role in reducing the risk of cancer. They further demonstrated that colon cancer incidence was inversely correlated with dietary vitamin D intake [9], and with serum 25(OH)D concentration in 1989 [10].

In 1999, the *Atlas of Cancer Mortality in the United States, 1950–1994* was published by the National Cancer Institute [138] (available a Google Books) with maps of incidence and/or mortality of about 37 types of cancer for two periods, 1950–1969 and 1970–1994. Deciles of mortality rate were displayed in shades of colors from dark blue for low rates to dark red for high rates. It was readily apparent that mortality rates for many types of cancer were highest in the northeastern states while they were lowest in the southwestern states. An article published in 2002 showed that solar UVB doses at the Earth’s surface in July 1992 were inversely correlated with mortality rates for 506 state economic areas in the 48 contiguous U.S. states (omitting AK and HI because solar UVB doses were at extreme vlues), but omitted data for the states bordering Mexico where *H. pylori* infection, an important risk factor for gastric cancer, is unusually common [139] and the high rates of gastric cancer reflected the high proportion of residents from Mexico. In a follow-up article, data were included for several cancer risk-modifying factors including alcohol consumption, Hispanic heritage, poverty, smoking, and urban/rural residence, and data were analyzed by state for white Americans [140] and later for black Americans [141]. Both of the articles for white Americans identified about 13 types of cancer; the rates of which were inversely correlated with solar UVB doses; and adjusting for the other known cancer risk-modifying factors did not significantly change those cancer-correlations with solar UVB doses.

Evidence relevant to the role of vitamin D in reducing cancer incidence and mortality rates was published recently [28]. The principal findings were reported for seven large ecological single-country studies of cancer incidence and/or mortality rates in relation to indices of solar UVB dose (latitude or UVB dose). It also included a novel study on cancer incidence with respect to occupation in Nordic countries which looked at lip cancer incidence less lung cancer incidence as a UVB exposure index [142]. From those data, over 30 types of cancer had their incidence rates examined in relation to solar UVB doses—inverse correlations with solar UVB dose indices being found for all but four types of cancer (Table 2 and Table 3; [28]). Since no effects of solar UVB exposure on cancer risks have been found other than vitamin D production, these results provide strong support for the role of vitamin D in reducing risk of cancers (other than cutaneous basal cell and squamous cell carcinomas).

Many observational studies have been reported that have examined serum 25(OH)D concentration and cancer incidence rates. Table 5 of that article [28] lists meta-analyses for all cancers, and for bladder, breast, colorectal, head and neck, liver, lung, ovarian, pancreatic, prostate, renal, and thyroid cancers. Nearly all meta-analyses have reported significant inverse correlations between serum 25(OH)D concentration and cancer incidence from prospective and CC studies apart from a finding of insignificance for ovarian cancer and a direct correlation for prostate cancer. As already suggested, [28], the findings for prostate cancer may be due to increased absorption of calcium and phosphorus from oral intake since one or both minerals are suggested risk factors for prostate cancer [143].

One of the problems with prospective observational studies is that serum 25(OH)D concentrations change over time, so that the longer the follow-up time, the lower the apparent benefit of higher baseline 25(OH)D concentration [28,40]. An example of this effect is shown in Figure 1 of that article [28] for a meta-analysis of prospective studies of colorectal cancer incidence vs. serum 25(OH)D concentration reported earlier [144], where for each 10 ng/mL increment in circulating 25(OH)D, colorectal cancer risk was 19% lower in women (RR = 0.81, 95% CI = 0.75 to 0.87) and 7% lower in men (RR = 0.93, 95% CI = 0.86 to 1.00). Since there is no a *priori* reason to suggest that serum 25(OH)D concentrations would have a different effect on cancer incidence for men and women, further investigations were undertaken. It was found that for men, the OR increased by 0.031/year while for women, the increase was 0.0081/year. The regression fit to the data as a function of follow-up time was OR = 0.74 for men and 0.77 for women. (See Figure 1 in [28].) An article by Visvanathan et al. conducted a pooled analysis of 17 prospective cohort studies of breast cancer incidence with respect to baseline serum 25(OH)D concentration in an effort to determine whether vitamin D reduces the risk of breast cancer [145]. All of the RRs were near 1.0 with no variation with respect to median follow-up time after blood draw for 25(OH)D concentration measurement, which ranged from 2.3 to 12.4 years. This is likely because of the potential of breast cancer to develop rapidly [146].

CC studies use data on many variables examined near the time of disease diagnosis to evaluate risk factors and usually find stronger correlations with health outcomes than do prospective studies. However, CC studies are generally considered inferior to prospective studies for two reasons: first, that the disease state may affect the variable factors studied [reverse confounding] and second, that the controls may not have been fully comparable. Furthermore, as discussed recently, [33], serum 25(OH)D concentrations can be lowered near the onset of acute inflammatory diseases such as COVID-19, but not usually in other disorders though obesity and diabetes lower 25(OH)D chronically through proven mechanisms [147]. Cancer is not an acute inflammatory disorder and inflammatory biomarkers are not raised in early-stage cancer [28]. As for the selection of controls, the propensity score matching approach is useful and may be more reliable than the usual randomized control matching [148]. The reason is that important variables that affect the outcome can be matched for both cases and controls. An example of propensity score matching in a vitamin D RCT used the following matching covariates: age, baseline 25 (OH)D, total energy intake, smoking, alcohol consumption, dietary fiber, saturated and unsaturated fat intakes, adiposity, and physical activity [149]. A comparison of CC studies with prospective studies of breast and colorectal cancer found that CC data gave RRs close to those expected from regression analysis of prospective observational study data [40].

A CC study of breast cancer incidence in Iran was reported in 2016 [150]. It included 135 cases from April 2013 to May 2014 along with 135 cases matched by 10-year age groups and menopausal status. Table 5 in [150] shows that the aOR for quartile 25(OH)D ≥ 29.5 ng/mL compared to the quartile ≤ 10.3 ng/mL was 0.26 (95% CI, 0.12–0.59). The aORs for the second and third 25(OH)D quartiles were 0.48 (95% CI, 0.22–1.04) and 0.59 (0.28–1.29), respectively. Since few such studies have been conducted in the Middle East, the importance of this study is that it found results similar to those found in Western countries.

RCTs are considered the best way to determine causality in medicine. However, as discussed earlier, vitamin D RCTs have been designed based on vitamin D dose as is the norm in pharmaceutical drug RCTs rather than being based on changes in serum 25(OH)D concentrations as is recommended for nutrients [32]. Unsurprisingly, therefore, very few have, as yet, reported vitamin D supplementation to reduce cancer risks (see Table 9 in [28]). The vitamin D RCT that had the best chance of finding a beneficial effect of vitamin D supplementation on cancer risks was the VITAL study [54]. It enrolled over 25,000 participants and supplemented half of them with 2000 IU/d vitamin D_3_. While vitamin D supplementation was not associated with cancer incidence when analyzed by intention to treat, cancer mortality rate, with omission of the data for the first, or first + second, years of supplementation, was inversely associated with cancer risks (hazard ratios, 0.79 [95% CI, 0.63 to 0.99] and 0.75 [95% CI, 0.59 to 0.96], respectively). In addition, secondary analyses revealed that for those with BMI < 25 kg/m^2^, cancer incidence was reduced [HR = 0.76 (95% CI, 0.63–0.90)]. The reason why vitamin D is more effective in reducing risk of cancer mortality than cancer incidence is, probably, that while there are many mechanisms driving cancer incidence there are few mechanisms of importance for reducing angiogenesis around tumors, and thereby reducing metastasis risk, apart from vitamin D status, as recently reviewed [33].

That vitamin D reduces risk of cancer in a causal manner has been evaluated using Hill’s criteria for causality [36]. Hills criteria were first used for cancer generally in 2009 [151] and later for breast cancer [152]. By 2009, only one RCT had found that vitamin D supplementation reduced risk of cancer incidence which was from Nebraska, USA [15]. Now, however, a secondary analysis from the VITAL study [54] provides further support for a role of vitamin D in reducing cancer risks as discussed above.

The estimate of reductions in cancer mortality rate for raising the minimum 25(OH)D concentration to 30 ng/mL is based on a meta-analysis of five vitamin D RCTs [153,154]. The estimate in [154] was a reduction of 13% (95% CI, 4–21%), based on a mean vitamin D supplementation dose, of 1000 IU/day. However, two of the RCTs used monthly bolus doses, which have been found to be ineffective in reducing the risk of non-skeletal health outcomes; one trial gave 400 IU/day and a second, 800 IU/day and in the VITAL study, 2000 IU/day [54]. The results were also based on the full length of each study. However, as reported in the VITAL study, a significant reduction in overall cancer mortality rate was found only when data for the first one or two years of data were omitted [54]. In analyses restricted to 153 deaths from cancer in patients with medical records or other adjudication of the cause of death beyond the NDI coding, the HR were 0.72 (95% CI, 0.52–1.00) over the total follow-up period and 0.63 (95% CI, 0.43 to 0.92) after the first 2 years were excluded. The mean baseline 25(OH)D concentration for those in the vitamin D treatment arm providing values was 31 ng/mL. Since the effect of a given vitamin D dose will be greater in those with lower 25(OH)D concentrations, it is reasonable to expect that the reduction in overall cancer mortality rates by raising the minimum 25(OH)D concentration in KSA and the UAE, where deficiency remains common, will be greater than the 37% (8–57%) found in the VITAL study.

## 11. Respiratory Tract Infection and Other Infectious Diseases

### 11.1. Influenza

Interest in the role of vitamin D in reducing risk of respiratory tract infections increased dramatically after Cannell and colleagues published a paper in 2006 hypothesizing that the rate of seasonal variation in epidemic influenza in winter being due to low solar UVB doses causing reduced vitamin D production [13]; that report referred to the paper by Hope-Simpson showing the same relationship in both the northern and southern hemispheres [155]. This hypothesis was quickly supported by an analysis of influenza and cold incidence in a vitamin D RCT conducted on African–American postmenopausal women in New York [14] and later in a vitamin D RCT in school children in Japan [17]. However, it was subsequently shown that absolute humidity was a more important determinant of the winter peak compared to vitamin D status for influenza [156], and also that cold-dry weather conditions were most important outside the tropics while humid-rainy conditions was the most important factor in the tropics [157]. A study in the Nordic countries found that temperature and UV dose were the major predictors of influenza rates in winter, with a lesser association with humidity [158]. A meta-analysis of ten trials including 4859 individuals found that supplementation with vitamin D significantly reduced the risk of influenza infections (*RR* = 0.78, 95% CI:0.64–0.95) [159]. There have not been any subsequent meta-analyses on vitamin D and influenza risks as of 7 February 2023.

There is also evidence that higher UV exposure and higher vitamin D status may reduce the risk of pneumonia. A study conducted in Philadelphia, USA involving 602 cases of invasive pneumococcal disease (IPD) [160] showed that IPD incidence was greatest in winter, peaking in week 51. After adjustment for seasonality, weekly incidence was found to be inversely associated with clear-sky UV index (IRR per unit increase in index: 0.70 [95% CI 0.54–0.91]). The effect of UV index was highest among the youngest people and decreased with age. Over shorter time scales only, an association was found between increases in ambient sulfur oxides and increased disease risks (incidence rate ratio = 1.73 (95% CI, 1.27–2.37)).

In a study in China of 163 older hospitalized patients, 49 suffering from pneumonia [161], levels of 25(OH)D, total cholesterol (TC), and low-density lipoprotein cholesterol (LDL-C) were lower (*p* < 0.05) in the pneumonia group and severe vitamin D deficiency was significantly more common in the pneumonia group (71.4 vs. 19.3%; *p* < 0.0001). Multivariate logistic regression showed that age and 25(OH)D levels were independent risk factors for pneumonia. Since 25(OH)D concentrations decrease rapidly during acute inflammatory diseases [162], the low 25(OH)D concentrations were to be expected but the effects on health outcomes of falls in serum 25(OH)D due to infection compared to those due to inadequate provision remain unknown.

### 11.2. Acute Viral Respiratory Tract Infections

A meta-analysis for double-blind RCTs of several forms of vitamin D supplementation conducted before 1 May 2020 was analyzed for acute respiratory tract infection rates (ARI) [163]. The authors identified 1528 articles, of which 46 RCTs (75,541 participants) were eligible for analysis. Protective effects of supplementation were observed in trials where vitamin D had been given in a daily dosing regimen (OR 0·78 [95% CI 0·65–0·94]; 19 studies), at daily dose equivalents of 400–1000 IU (0·70 [0·55–0·89]; ten studies), and in participants aged 1–16 years at enrolment (0·71 [0·57–0·90] in 15 studies).

### 11.3. COVID-19

It was suggested as early as April 2020 that boosting vitamin D status could reduce the risk of SARS-CoV-2 infection and progression to COVID-19 [164]; analogies were made with respect to the pneumonia associated with pandemic influenza [165] and with influenza and other acute respiratory viral illnesses. The proposed mechanisms included reduced viability of the SARS-CoV-2 through induction of human cathelicidin (LL-37) and reduced production of proinflammatory cytokines and reduced risk of a cytokine storm. A large number of studies have evaluated this hypothesis.

An observational study from the UAE investigated COVID-19 severity and death with respect to vitamin D status, [166] using data for 464 patients who were admitted for COVID-19 to two of the main hospitals in Abu Dhabi and Dubai. Serum 25(OH)D concentrations were determined from blood drawn at the time of admission. The aOR for COVID-19 severity with respect to 25(OH)D concentration < 12 ng/mL vs. >12 ng/mL was 1.76 (95% CI, 1.19–2.61) and the aOR for mortality with respect to 25(OH)D concentration < 12 ng/mL vs. >12 ng/mL was 2.58 (95% CI, 1.01–6.62). Higher age was associated with an independent increase in risk of 7% for both outcomes.

One of the first retrospective studies carried out in KSA during the global lockdown involved 439 Saudi adults admitted for COVID-19 and showed that having a serum 25(OH)D < 12.5 nmol/L vs. >12.5 nmol/l was a significant predictor of mortality [aHR 7.0 (CI 1.7–28.2); *p* = 0.007] [167]. An RCT of 2-weeks duration was conducted a year later in a Saudi setting, this time for COVID-19 patients with vitamin D deficiency on admission and found that daily doses of 5000 IU of D_3_, which exceeded local intake guidelines, improved recovery times [168].

Again, it must be stressed that serum 25(OH)D concentrations can be lowered by acute inflammatory illness [162] and that COVID-19 is one such disease. Thus, observational studies of COVID-19 with 25(OH)D concentration measured near the time of diagnosis can be considered useful in determining outcome but not for determining the risks of infection with this virus.

An observational study of COVID-19 outcomes with respect to serum 25(OH)D concentration for vitamin D supplemented patients compared to unsupplemented patients was reported from Barcelona [169]. In Barcelona, most vitamin D is obtained by prescription and records are kept of who has received vitamin D. Since cholecalciferol and calcifediol [25(OH)D] cost about the same there, both are used. Cases and controls were matched using propensity score matching. Diagnosis of COVID-19 was made between 25 February to 30 April 2020 while mortality date was obtained between 24 February and 15 May 2020. For those supplemented with cholecalciferol and with achieved 25(OH)D concentrations > 30 ng/mL compared to unsupplemented controls with 25(OH)D concentrations < 20 ng/mL, the aHR for SARS-CoV-2 infection was 0.60 (95% CI, 0.57–0.77), the aHR for severe COVID-19 was 0.72 (95% CI, 0.52–1.00), and the aHR for COVID-19 mortality was 0.66 (95% CI, 0.46–0.93). For those supplemented with calcifediol, the aHR for SARS-CoV-2 infection was 0.69 (95% CI, 0.61–0.79), the aHR for severe COVID-19 was 0.61 (95% CI, 0.46–0.81), and the aHR for COVID-19 mortality was 0.56 (95% CI, 0.42–0.76) [169].

A study of the effect of vitamin D supplementation on COVID-19 infection and mortality rates for American veterans treated by US Department of Veterans Affairs health care facilities in 2020 was reported recently [170]. Records were reviewed for 66,432 patients treated with prescribed vitamin D_2_ and 398,996 patients treated with prescribed vitamin D_3_ in the period prior to the COVID-19 pandemic (1 January 2019 to 31 December 2020). That study also included patients treated from March 1 to 31 December 2020, but only if they had also been vitamin D supplemented in the earlier period. Those treated earlier with vitamin D developed COVID-19 infections rates of 28% and 20%, respectively, [(D_3_ HR = 0.80, [95% CI 0.77, 0.83]), D_2_ HR = 0.72, [95% CI 0.65, 0.79)] vs. untreated individuals. The overall HR for mortality within 30 days of diagnosis of COVID-19 was 0.68 (95% CI, 0.59–0.75), irrespective of season. They also noted that after controlling for serum 25(OH)D concentrations, veterans receiving higher dosages of vitamin D obtained greater benefits from supplementation than veterans receiving lower dosages. Thus, for example, veterans with 25(OH)Ds between 0 and 19 ng/mL exhibited the largest decrease in COVID-19 infection rates following supplementation and Black veterans showed greater COVID-19 risk reductions with supplementation than White veterans. The authors stated that “When we extrapolate our results for vitamin D_3_ supplementation to the entire US population in 2020, there would have been approximately 4 million fewer COVID-19 cases and 116,000 deaths would have been avoided.”

A recent article has shown that regardless of the severity of COVID-19 at the time of hospital admission, low serum 25(OH)D concentrations predicted poor outcomes [171]. The concentration of serum 25(OH)D at the time of admission is influenced by the concentration prior to infection and the degree of inflammation induced by fighting the disease [162]. There could be various reasons for the differences in the severity of the disease at the time of admission, such as varying symptom thresholds among individuals who seek admission to hospitals. These findings provide further evidence for the potential role of vitamin D in mitigating the severity of COVID-19.

Calcifediol has been used to treat COVID-19 patients in Spain. The benefits of using calcifediol rather than cholecalciferol are that serum 25(OH)D concentrations can be increased rapidly in hours rather than days [172] and that adverse effects of large bolus doses of D_3_ can be avoided, which is desirable since very large bolus doses are not as effective as daily dosing [173]. The first such study was reported in 2020 [174] on 76 consecutive patients hospitalized with COVID-19 who all received ‘best available therapy’ as standard. Eligible patients were allocated at a ratio of 2:1 to calcifediol versus no calcifediol by electronic randomization on the day of admission (oral calcifediol dosage being 0.532 mg). Patients in the calcifediol treatment group continued on oral calcifediol (at 0.266 mg) on days 3 and 7, and then weekly until discharge or ICU admission. Outcomes of effectiveness included rate of ICU admission and death rates. Of 50 patients treated with calcifediol, one required admission to the ICU (2%), while of 26 untreated patients, 13 required ICU admission (a reduction from 50% to 2% in ICU admissions). The overall multivariate Risk Estimate OR for ICU admission in patients with calcifediol treatment vs without calcifediol treatment (adjusted for hypertension and T2DM) = 0.03 (95% CI: 0.003–0.25). Of the patients treated with calcifediol, none died, and all were discharged without complications. The 13 patients not treated with calcifediol, who were not admitted to the ICU, were all discharged but of the 13 unsupplemented patients admitted to the ICU, two died and the remaining 11 were discharged.

In summary, there is moderate evidence that higher 25(OH)D concentrations reduce the risk of SARS-CoV-2 infection and strong evidence that they reduce the risk of severe COVID-19. The evidence regarding intact parent vitamin D_3_ in treating COVID-19 is only moderate. The evidence for benefit with calcifediol [25(OH)D] treatment, however, is encouraging and warrants further investigation. It appears reasonable that vitamin D should be given at the first symptoms of infection or disease in combination with ‘best treatment’ as an adjuvant measure in order to improve the immune response, while the available data suggests that serum 25(OH)D concentrations > 50 ng/mL compared to <20 ng/mL at the population level over time could halve COVID-19 risks [175].

## 12. Autoimmune Diseases

There are a number of autoimmune diseases for which there is evidence that better provision of vitamin D reduces risk. Observational studies in the KSA and UAE and elsewhere have found lower 25(OH)D concentrations associated with increased incidence and/or prevalence based on observational studies for disorders including: ankylosing spondylitis [176], inflammatory bowel disease [177], multiple sclerosis [178], rheumatoid arthritis [179,180] systemic lupus erythematous [181], T1DM [182,183], and vitiligo [184].

A recent article reported that vitamin D_3_ supplementation at 2000 IU/d in an RCT reduced the risk of combined autoimmune diseases [185]. 25,871 participants were enrolled and followed for a median of 5.3 years, mean baseline age = 67.1 years. For the vitamin D arm, 123 participants in the treatment group versus 155 in the placebo group developed a confirmed autoimmune disease [HR = 0.78 (95% CI, 0.61–0.99)]; the rate of development of autoimmune disease was very similar between the vitamin D and placebo arms over the first three years, after which autoimmune disease development rates increased more rapidly in the placebo arm. When the first two years of follow up were excluded from analysis the HR for new autoimmune disease = 0.61 (95% CI, 0.43–0.86) with supplementation. The largest effect was seen for ‘confirmed’ and ‘probable’ rheumatoid arthritis: 15 vs. 24 cases and 18 vs. 27 cases in the vitamin D treatment and placebo arms, respectively.

Viral infections are an important risk factor for autoimmune disease. A review of a total of 24,117 cases of incident RA (mean age 54.7 years; 18,688 [77.5%] women) found that respiratory viral infections in the population were associated with a higher incidence of RA over time, an effect peaking 6 or 7 weeks after infection [186]. The progression of multiple sclerosis (MS) is also triggered by certain environmental factors, including viral infections [187]. The most important viruses that affect MS risks are Epstein–Barr virus (EBV), human herpes virus 6 (HHV-6), human endogenous retrovirus (HERV), cytomegalovirus (CMV), and varicella zoster virus (VZV). These viruses all have latent stages that allow them to escape immune detection and to reactivate after exposure to various stimuli. Furthermore, viral tropism for CNS and immune system cells is likely to explain their potentially damaging neuroinflammatory effects [187].

Vitamin D status also affects the severity of autoimmune disease and a recent review noted that seasonal variations in onset, severity, and progression of rheumatoid arthritis were significantly, and inversely, correlated with serum 25(OH)D concentrations prospectively [188].

Experimental work has revealed a mechanistic basis for the contribution of vitamin D inadequacy to the pathogenesis of inflammatory bowel disease (IBD). The vitamin D/VDR complex is involved in the regulation of innate and adaptive immune responses to pathogenic threats by acting as an immunomodulator and can alleviate inflammation in experimental models of IBD and in patients with IBD, through promotion of intestinal wall homeostasis. Vitamin D has been associated with the promotion of antimicrobial peptide secretion, down-regulation of dendritic cell activity, induction of tolerogenic rather than pro-inflammatory T-cell differentiation and function, and increased production of anti-inflammatory cytokines, highlighting its potential therapeutic value for IBD [177]. Similar benefits could also be expected for other inflammatory bowel disorders since experimental studies have shown that hormonally active vitamin D [1,25-dihydroxyvitamin D (calcitriol)] stimulates immunologic activity in both the innate and the adaptive immune systems and promotes endothelial membrane stability in the gastrointestinal tract. Low levels of serum 25(OH)D are associated with increased risks of developing immune-related diseases. However, different disease outcomes are observed aftertreatment with vitamin D because high inter-individual differences are present due to the complex gene expression in human peripheral blood mononuclear cells. Thus, the optimal level, or levels, of serum 25(OH)D for autoimmune disease risk reduction are not yet clear. The current recommendation is to increase vitamin D intake and have enough sunlight exposure to achieve serum 25(OH)D concentrations at or above 30 ng/mL (75 nmol/L), though values of about 40–60 ng/mL (100–150 nmol/L) may be needed to promote optimal autoimmune risk reductions with vitamin D [189].

Another review has discussed the role of epigenetics (a series of gene regulatory effects that do not disrupt genetic DNA sequences) in the etiology of autoimmune disease [190]. The main epigenetic mechanisms considered to play a major role in both health and disease are DNA methylation, histone modifications, and altered profiling of non-coding RNAs. When the fragile balance between these simultaneously occurring phenomena is disrupted, the risk of pathology increases; those authors aimed to review the literature suggesting that vitamin D is one of the more important nutrients potentially capable of modulating the course of disease in various autoimmune disorders [190]. Crohn’s disease, for example, may be a condition where adequate provision of vitamin D can reduce the risk of relapse [191].

The importance of maintaining sufficient vitamin D concentrations in maternal blood during pregnancy, as well as in the early years of life, for avoiding later offspring ill health due to inappropriate epigenetic changes in deficiency has also been emphasized. An example is atopic eczema at age 4 years [192]. Another example is increased childhood adiposity is associated with higher maternal adiposity and low maternal vitamin D status [193].

## 13. Pregnancy and Birth Outcomes

Having optimal maternal and neonatal vitamin D provision (circulating 25(OH)D concentrations) prior to, during, and after pregnancy is considered critical for pregnancy outcomes. However, by 2019, the significance of optimal vitamin D status during pregnancy was still not universally accepted [194,195] because the few large RCTs conducted to date have generated conflicting evidence on vitamin D supplementation in improving perinatal outcomes. However, as we now understand, most vitamin D RCTs conducted to date have been inappropriately designed, conducted, and analyzed [33,196], so that lack of support from earlier RCTs where, for example, achieved vitamin D status was not examined should not be a major concern since the peer-reviewed literature does provide good evidence for promotion of reproductive health by adequate vitamin D status. This includes that vitamin D may improve fertility for both males and females [197,198], that vitamin D promotes normal development of the fetus by ensuring the necessary epigenetic changes take place [199,200] that vitamin D reduces the risk of preterm birth, of small for gestational age births and of stillbirth as well as of pre-eclampsia with its associated increase in maternal and neonatal risks [201], that vitamin D reduces maternal morbidity and mortality [202,203], and reduces the risks of gestational diabetes [204]. Intact (unprocessed) vitamin D is needed by nursing infants [205] and repletion by the time of birth avoids the risks of neonatal hypocalcaemic status epilepticus [206] and of hypocalcaemic cardiomyopathy [207] both of which have high mortality rates unless they are correctly diagnosed and treated very rapidly.

In KSA, an extremely high prevalence of maternal (85%) and neonatal (88%) vitamin D deficiency (<20 ng/mL) has been documented [208] and associated with a GDM risk that is three times higher than in those who do not have vitamin D deficiency [209].

An informative RCT of vitamin D in pregnancy has been conducted in Iran [210]. It involved 800 pregnant women in two Iranian cities. Women with a moderate [25(OH)D, 10 to 20 ng/mL] and severe [25(OH)D, <10 ng/mL] deficiency were randomly divided into four subgroups and received vitamin D_3_ until delivery. Supplementation dose was determined by baseline 25(OH)D concentration and by group, with four groups for each of two 25(OH)D concentration ranges, <10 ng/mL and 10–20 ng/mL, 50,000 IU/week to two intramuscular doses of 300,000 IU followed by 50,000 IU/month. After supplementation, only 2% of the women in the control group met the sufficiency level (>20 ng/mL) vs 53% of the women in the screening and treatment arm. The screening site had 900 pregnant women living in Madjed-Soleyman while the treatment arm had 900 pregnant women living in Shushtar. Most of the baseline characteristics such as age, 25(OH)D concentration and blood pressure were non-significantly different between the two groups, [most *p* values above 0.50 with two of 0.07, for marriage age and 25(OH)D concentration [23 ng/mL (IQR, 21–27 ng/mL) and 22 ng/mL (IQR, 21–24 ng/mL) in the two groups]. Adverse pregnancy outcomes, including preeclampsia, gestational diabetes mellitus, and preterm delivery, were significantly decreased, by 60%, 50%, and 40%, respectively, with supplementation. While differences in confounding factors between the two locations cannot be ruled out, it seems unlikely that they would explain the huge differences in the outcome measures. One group with baseline 25(OH)D concentrations between 10 and 20 ng/mL and two groups with a serum 25(OH)D < 10 ng/mL were given an initial intramuscular vitamin D_3_ injection of 300,000 IU in addition to monthly 50,000 IU maintenance injections (given as being most likely to ensure compliance and to achieve vitamin D sufficiency by the time of delivery). One of the important factors in that trial was that the participants who were not supplemented with vitamin D had a mean baseline 25(OH)D concentration of 11 ng/mL (7–16 ng/mL), which was virtually unchanged at delivery. Thus, this RCT satisfied the guidelines for nutrients outlined by Heaney [32]. However, some confounding due to problems induced by large bolus dosing cannot be excluded [210].

A cross-sectional observational study conducted in Iran aimed to determine the thresholds for serum 25(OH)D) concentrations necessary for reducing adverse pregnancy outcomes, including preterm labor, preeclampsia (PE), and gestational diabetes mellitus (GDM), using a generalized additive model [211]. It used the data of 1763 pregnant women, whose serum vitamin D status during the third trimester of pregnancy was available. The concentrations of 25(OH)D within which there were high, moderate, or low risks of GDM were ≤16, 16–26, and >26 ng/mL, respectively. Similarly, the ranges of serum 25(OH)D concentrations associated with high, moderate, and low risks of preterm delivery were ≤15, 15–21, and >21 ng/mL, respectively. Finally, the corresponding values for the high, moderate, and low risk of pre-eclampsia [PE] were ≤15, 15–23, and >23 ng/mL, respectively. Those models were well-calibrated, using the Hosmer-Lemeshow test. Results using an adjusted generalized linear model showed a significant trend for increasing risk of adverse pregnancy outcomes with lower baseline 25(OH)D concentrations. While those authors recommended that in the preconception period, a 25(OH)D concentration of >15 ng/mL is adequate for the prevention of adverse pregnancy outcomes, the overall data available suggests that >30 ng/mL would be a more appropriate target level to ensure optimal pregnancy outcomes.

An observational study in Boston found that risk of primary Cesarean-section delivery was inversely correlated with serum 25(OH)D concentration [212]. A total of 253 women were enrolled between 21 March 2005, and 20 March 2007. In the study, 67 had cesarean sections, of whom 43 had a primary cesarean section. The aOR for primary cesarean section for a baseline maternal 25(OH)D <15 ng/mL compared to >15 ng/mL was 3.84 (95% CI, 1.71–8.62 0.001).

A meta-analysis of Cesarean section in gestational diabetes mellitus patients supplemented with vitamin D was reported in 2020 [213]. The relative risk of Cesarean section for vitamin D supplementation vs. placebo based on five studies was 0.61 (95% CI, 0.44–0.83). Based on the same five studies, the RR for macrosomia was 0.31 (95% CI, 0.13–0.72), no doubt associated with the reduced risk of GDM.

An interventional observational study regarding vitamin D supplementation and risk of preterm birth was conducted at the Medical University of South Carolina [214]. A total of 1064 women of various ethnic backgrounds were included in the study. At their first prenatal visit, serum 25(OH)D concentration was measured and they were given a free bottle of 5000 IU vitamin D_3_ capsules and counseled on how to achieve 25(OH)D > 40 ng/mL. Serum 25(OH)D concentration was measured several times during the study and the value closest to delivery was used for analysis. The mean rate of preterm delivery increased from 36.8 weeks with a maternal 25(OH)D of 5 ng/mL to 38.3 weeks at 25 ng/mL and 39 weeks at 90 ng/mL. The OR for indicated preterm birth for 25(OH)D concentration > 40 ng/mL vs. <20 ng/mL was 0.39 (95% CI, 0.20–0.76).

## 14. Skeletal Muscles and Vitamin D

The significance of vitamin D in human muscles cannot be emphasized enough. The following is a summary of essential discoveries from previously published reviews, arranged in chronological order. The importance of vitamin D for athletic performance was outlined by Cannell et al. in 2009 [215]. They pointed out that Germans used UVB lamps to produce vitamin D and improve athletic performance in the 1950s, and that athletic performance has seasonal variations associated with serum 25(OH)D concentrations affected by solar UVB. Vitamin D increases the size and number of Type II (fast twitch) muscles. Hamilton reviewed the topic in 2010 [216]. He noted that vitamin D receptors were discovered in rat myoblast cells in 1985 [217], thus showing that vitamin D controls muscles through genomic actions in addition to those by calcium affecting both contractile and relaxation properties [218]. Vitamin D also regulates insulin-like growth factor-1 (IGF-1) [219]. IGF-I induces proliferation, differentiation, and hypertrophy of skeletal muscle [220].

A study by Jastrzebska et al. [221], aiming to investigate the relation between 25(OH)D concentrations and athletic performance among young Polish soccer players (N = 24) throughout the training cycle across different seasons during lockdown, revealed significant changes in 25(OH)D concentrations with season. The highest concentrations were observed at the end of the summertime in September and August, while the highest concentrations were observed during the low sunlight periods in December. Moreover, the results demonstrated a significant correlation between 25(OH)D concentrations and sprint times on the distances of 10 m and 30 m, where athletes with greater concentrations of 25(OH)D in performed better and had shorter sprint times. Additionally, there was also a significant improvement in the jump test. There was no significant correlation between 25(OH)D concentration and other physical fitness measurements.

Insulin resistance (IR) in older men results in lean mass loss [222]. In muscle, vitamin D reduces IR by reducing over-production of FOXO1 and is dependent on VDR activation of intracellular insulin signaling pathways, for example, through enhancement of Insulin receptor substrate-1 (IRS1) and VDR production in muscle tissue [24].

In 2012, Bischoff-Ferrari discussed the role of vitamin D in reducing the risk of falls [223]. Based on an analysis of results from RCTs, vitamin D doses between 700 and 1000 IU/day significantly reduced the risk of falling [OR = 0.66 (95% CI, 0.53–0.82)] [224]. In addition, achieving serum 25(OH)D >24 ng/mL significantly reduced falling [pooled RR = 0.77 (95% CI, 0.65–0.90)] [225].

An important problem in old age is sarcopenia, cachexia, and muscle atrophy [226]. The loss of muscle mass is around 0.8%/year in the forties rising to 1.5%/year in the 60s [227]. Vitamin D supplementation may reduce the progression of sarcopenia [228]. Observational and mechanistic studies suggest that vitamin D supplementation might be an effective way to prevent and treat sarcopenia [229]. The definition of cachexia that emerged from a conference in 2006 was: “cachexia is a complex MetS associated with underlying illness and characterized by loss of muscle with or without loss of fat mass. The prominent clinical feature of cachexia is weight loss in adults (corrected for fluid retention) or growth failure in children (excluding endocrine disorders). Anorexia, inflammation, insulin resistance, and increased muscle protein breakdown are frequently associated with cachexia” [230].

A review published in 2019 covered the mechanisms of vitamin D on skeletal muscle function: oxidative stress, energy metabolism, and anabolic state [231]. Vitamin D deficiency decreases oxygen consumption and induces disruption of mitochondrial function. Vitamin D deficiency may also contribute to the development of muscle atrophy through a pathway causing protein degradation.

## 15. Obesity and Vitamin D

Several epidemiological studies, including large cohort studies such as the National Health and Nutrition Examination Survey (NHANES) and Framingham Heart studies, have reported an association between obesity measures (including increased BMI and increased waist circumference) and low serum 25(OH)D concentrations [232,233,234,235]. Individuals who suffer from obesity, BMI > 30 kg/m^2^, consistently have lower serum 25(OH)D concentrations; an observation that holds true across ages, ethnicities, genders, geographies and cultures where serum 25(OH)D is inversely correlated with body weight, BMI, and fat mass [236].

These inverse relationships between measures of obesity and vitamin D status (serum 25(OH)D) in individuals with obesity have been explained as being due either to vitamin D sequestration by the various fat or fat-containing body organs in individuals with obesity [235], or by simple dilution into increased fat masses, the “volumetric dilution hypothesis” [237], or by some combination of the differences in the expression and activity of the vitamin D hydroxylase enzymes seen in obese versus lean (normal weight) individuals [235], especially in the liver and adipose tissues [235,238], in the adverse behavioral or cultural lifestyle habits adopted by individuals with obesity, including reduced outdoor activity and avoidance of sun exposure, or in the secondary biochemical complications such as elevated circulating parathyroid hormone (PTH) [239] and/or due to other as-yet unrecognized factors.

Wortsman et al. [235] were the first to suggest that individuals with obesity compared to lean (normal weight) individuals consistently gave lower responses to UV-B and achieved lower serum 25(OH)D concentrations than lean individuals, despite obese individuals having a greater body surface area to support vitamin D’s synthesis when stimulated with Ultraviolet B (UV-B) exposure. This lower serum 25(OH)D concentration following UV-B exposure was hypothesized to be due to vitamin D metabolites being sequestered and tightly locked into adipose tissues and other fat-containing organs, making it difficult for it to be released back into the blood stream. Possible mechanisms of locking/binding/sequestration, however, have not yet been reported [235].

Recently, differences in adipose and hepatic vitamin D-hydroxylating enzymes expression and activity between obese and lean individuals have been found, suggesting that 25(OH)D production and metabolism may be altered in obesity [238] which warrants further investigation. These findings are supported by emerging evidence from studies on obese rodents fed high-fat diets, where reduced 25-hydroxylating enzyme function and expression was found [9]. These adverse effects of obesity on 25(OH)D concentrations, bioavailability and activity may also be associated with the quantity and quality of the high-fat nutritional intake often seen with unhealthy nutritional and behavioral choices made by individuals with obesity [240].

Drincic et al. [237] hypothesized that the reason for the reduced serum 25(OH)D seen in individuals with obesity was due to a simple ‘volumetric dilution’. Having studied individuals spanning a range of body weights between 41 and 166 kg and of BMIs between 16.5 and 61.2 kg/m^2^, they proposed that the inverse correlation of 25(OH)D with these measures of obesity reflected simple distribution into larger fat masses with greater volumetric dilution. It is now known that people who are overweight need ×1.5 as much and people who suffer from obesity need 2 to 3 times as much vitamin D than leaner, lower weight individuals to achieve a comparable vitamin D status [241,242]. Consequently, vitamin D supplementation cannot be managed as ’one size fits all’ and the knowledge of the increased dose requirements of overweight and obese individuals should be considered in public health planning. Progress is being made to unravel the metabolic mechanisms of vitamin D metabolism in adipose tissues of individuals with obesity [243,244].

In a systematic review with meta-regression analysis of 23 studies, Pannu et al. showed that reductions in body weight, specifically percentage fat mass, in obese individuals resulted in a marginally significant increase in circulating 25(OH)D. These findings were taken to support the ‘volumetric dilution of hypothesis’ by Drincic et al. Nonetheless, the authors’ findings showed that the regression analysis slopes showed a smaller increase than expected in serum 25(OH)D following weight loss. These findings suggested that in addition to the ‘volumetric dilution’, a potential metabolic inactivation of 25(OH)D occurred in adipose tissues of obese individuals which could explain the greater reduction in released serum 25(OH)D upon fat mass and weight loss in these individuals; such inactivation could be different in different fat compartments in the body and warrants further investigation [245].

A study by Mason et al. (2011) showed that a loss of fat mass of more than 15% results in an average of 7.7 ng/mL increase in serum 25(OH)D in women [246]. In a bi-directional MR analysis study of 21 adult cohorts of up to 42,024 participants, Vimaleswaran et al. (2013) showed that each increase of 1 kg/m^2^ in BMI was associated with 1.15% lower circulating 25(OH)D. A 10% higher genetically instrumented BMI was associated with 4.2% lower 25(OH)D concentration (instrumental variables (IV) ratio: 24.2 [95% CI, 27.1–21.3], *p* = 0.005) but there was no association of genetically instrumented 25(OH)D with variations in BMI. The findings of this bi-directional MR analysis strongly suggest that a higher BMI leads to lowering of the serum 25(OH)D, while any effects of lower 25(OH)D on BMI appear to be minimal or absent. Those results, moreover, suggest that interventions that help reduce excessive BMI can be expected to ameliorate vitamin D deficiency [55]. In contrast, in line with the findings on bi-directional MR analyses on this association mentioned above, vitamin D supplementation has no effect on weight or fat mass loss [247]. In a later meta-analysis, Pannu et al. showed that a loss of approximately 10 kg without vitamin D supplementation could increase 25(OH)D concentrations by up to 6 nmol/L [245].

The important question is whether obesity-induced reductions in 25(OH)D concentrations have direct causative effects on adverse clinical outcomes associated with vitamin D deficiency in individuals with obesity. For bone health and turnover, it would be expected that lower 25(OH)Ds would lead to lower calcium absorption and lower bone mineral density (BMD) in otherwise healthy adults. However, in adults with obesity, despite the inverse association of obesity with lower serum 25(OH)D values, this does not translate into lower bone density or bone loss in obese adults. Indeed, some studies show that obese adult individuals may have higher bone density, higher BMD, thicker and denser cortices, and greater trabecular numbers than lean individuals [248]. However, in children and the elderly this obesity-evoked reduction in serum 25(OH)D has negative clinical outcomes related to weaker BMD, with increased fractures in children and the elderly [249,250,251]. As for other body systems, obesity-induced reduction in 25(OH)D clearly associates, often causally, with increased risks of many other comorbidities and conditions including cancer, metabolic syndrome, type 2 diabetes mellitus, cardiometabolic abnormalities, blood pressure, depression, autoimmune diseases, and others, as mentioned elsewhere in this manuscript and in the literature [252,253,254,255].

In the UAE and KSA, as with other Arab countries within the Middle East, obesity is continuously on the increase. Alongside the indigenous UAE and KSA Gulf Arabs living in their countries, expatriates of several nationalities, from different ethnic and religious backgrounds, have also settled there during the last five decades. Given the advanced rates of economic growth of these countries over the past 50 years, and the influx of expatriate worker migrants, drivers of obesity in the UAE and KSA tend to be complex. These include dietary shifts away from traditional foods to Western energy-dense diets, socio-economic drivers to consuming carbohydrate-rich but nutrient poor foods in poorer middle-income migrants, poor or unregulated nutritional education and unhealthy behavioral eating habits, multi-factorial anxiety and stress in high-powered vocations, lower levels of exercise especially in women, avoidance of outdoor physical activity during the prolonged months of very hot weather, and many other more recent lifestyle-related factors that have changed over the last 50 years of modern history [45]. Such a high prevalence of obesity is mirrored in several neighboring Middle Eastern countries, as reported in 2020 by the World Obesity Federation (https://www.worldobesity.org/, accessed on 9 February 2023).

Several studies conducted in the KSA and the UAE have investigated the connection between obesity, the ensuing vitamin D deficiency, and associated comorbidities. They confirmed the inverse association between obesity and circulating 25(OH)D levels, and have suggested an increased risk of vitamin D deficiency-associated comorbidities such as diabetes mellitus, depression, non-alcoholic fatty liver disease (NAFLD), reduced BMD, and cancer amongst other health concerns [86,256,257,258,259,260,261]. Given the alarming levels of obesity in KSA and the UAE and the further predicted rises in obesity rates in the future, it is important to implement, as a matter of urgency, improved public health measures that can ensure vitamin D sufficiency across populations including individuals with obesity. This should be coupled with measures aimed at reducing pandemic obesity rates through appropriate measures. Such practices should aim to increase serum 25(OH)D concentrations to above 30 ng/mL if improved health outcomes are to be expected.

## 16. Summary and Conclusions

In this narrative review we summarized the evidence on correcting vitamin D deficiency in relation to reducing adverse clinical outcomes for a variety of diseases. We also use data on disease prevalence and vitamin D status in KSA and the UAE to estimate the potential health benefits to be expected from raising 25(OH)D concentrations to a minimum of 30 ng/mL and these included expected reductions, for example, of 25% for MI incidence, 35% for stroke incidence, 20 to 35% for CVD mortality and possibly even more for prevention of type 2 diabetes. From VITAL study [54], it is estimated that cancer mortality rates could be reduced by 35%.

We have to stress that some of our estimated health benefits for increasing vitamin D status are based on factors that include various assumptions such as the inference of causality from MR and observational study data, and that our expert opinion-based selection of studies are not solely derived from systematic reviews and may, therefore, be at risk of bias. Additional limitations of our data interpretation are those inherent to the nature of the studies included in our review and potentially include various types of bias and confounding. Our conclusions and estimated calculations should, therefore, be interpreted in the light of these limitations. Nevertheless, we aimed to include the best available evidence up to 11 February 2023 to provide guidance for developing public health measures to reduce the health burden of vitamin D deficiency in KSA and the UAE, because substantial reductions in adverse health outcomes in Saudi Arabia and the UAE are likely to be achieved by raising the minimum serum 25(OH)D concentration to above 30 ng/mL. The U.S. Endocrine Society [4] and the consensus from vitamin D conferences in Poland [29,262] recommend daily intakes up to 2000 IU/d vitamin D_3_, while reviews from the U.S. Institute of Medicine in 2011 [3] and from the UK both demonstrate, and affirm, that 4000 IU/d is safe and results in greater health benefits. Food fortification with vitamin D can be effective, using a variety of foods including fats, dairy produce, and flour-based products [263,264] though supplementation is usually required in addition for those at high risk of deficiency such as the elderly, those with indoor life styles, shift workers, pregnant women, the obese, diabetics, dark-skinned individuals [255,265], and vegan/vegetarians [266]. Increased exposure to solar UVB can also be advantageous, as long as the solar elevation angle lies below 45 degrees [38] and sunburn is avoided, (e.g., by following the ’shadow rule’ [267]). While concern about erythema and skin cancers is often warranted, raising serum *25*(OH)D concentrations above 40 ng/mL can help reduce such risks; in part because skin is able to both produce and regulate vitamin D production, as well as to convert D_3_ into the 25(OH)D_3_ metabolite and then to 1,25-dihydroxyvitamin D_3_, (calcitriol), in situ, as do many other tissues [268], which promotes many protective mechanisms.

Testing 25(OH)D concentrations regularly would be beneficial, especially for those at greatest risk of vitamin D deficiency [255] when used as part of an ongoing audit of measures taken to avoid vitamin D deficiency [255]. It is, however, doubtful whether population-wide screening for vitamin D deficiency is cost-effective [269]. Programmes need to be developed for public health measures to ensure adequate vitamin D provision in various regions of the Middle East. These measures need to be practicable and acceptable to residents, as well as being confirmed as successful on audit [270]. To achieve these aims, committees or working parties could be established comprising appropriate health officials, medical doctors, nutritionists, and local community representatives. These parties should meet to review local evidence and agree on regional guidelines that will ensure effective measures are provided in an acceptable manner. In view of the findings reported in this review, it is hoped that programmes can be developed in the KSA and UAE that can achieve adequate vitamin D provision at the population level.

## Figures and Tables

**Table 1:** Mortality rates in 2016 for KSA and the UAE [41].

Outcome	KSA M *	KSA, F *	UAE, M *	UAE, F *
All-cause	777	608	604	511
IHD	219	154	182	144
Stroke	86	76	74	67
Cancer	64	57	55	62
Kidney disease	50	41	34	27
Alzheimer’s disease	46	44	34	40
Respiratory	46	42	24	18
Diabetes	30	25	39	40

(*) deaths/100,000/year; F, female; IHD, ischaemic heart disease; M, male.

**Table 2:** Estimate of reduction in MI incidence rate in the UAE by raising minimum 25(OH)D concentration to 30 ng/mL using 25(OH)D data from UAE [85] and results from a U.S. veterans study [80].

Lower 25(OH)D (ng/mL)	Upper 25(OH)D (ng/mL)	% in Lower Range in UAE	HR Lower (95% CI)	Reduction for 25(OH)D = 30–40 ng/mL (95% CI)	MI Reduction in the UAE (95% CI)
<20	>30	64	0.73 (0.55 to 0.96)	−0.27 (−0.45 to −0.04)	−0.17 (−0.29 to −0.03)
20–30	>30	22	0.65 (0.49 to 0.85)	−0.35 (−0.51 to −0.15)	−0.08 (−0.11 to −0.03)
					−0.25 (−0.40 to −0.06)

**Table 3:** Estimate of reduction in MI incidence rate in the KSA by raising minimum 25(OH)D concentration to 30 ng/mL using 25(OH)D data from [86] and results from a U.S. veterans study [80].

Lower 25(OH)D (ng/mL)	Upper 25(OH)D (ng/mL)	% in Lower Range in KSA	HR Lower (95% CI)	MI Reduction in the KSA (95% CI)
<20	>30	76	0.73 (0.55 to 0.96)	−0.21 (−0.34 to −0.03)
20–30	>30	20	0.65 (0.49 to 0.85)	−0.07 (−0.10 to −0.03)
				−0.28 (−0.44 to −0.06)

**Table 4:** Estimate of reduction in stroke incidence rate in the UAE by raising minimum 25(OH)D concentration to 30 ng/mL using 25(OH)D data from [85] and results from a U.S. study [84].

Lower 25(OH)D (ng/mL)	Upper 25(OH)D (ng/mL)	% in Lower Range in UAE	HR (95% CI)	1/HR (95% CI)	Stoke Reduction in the UAE (95% CI)
<20	20–30	64	1.85 (1.17–2.93)	0.54 (0.34 to 0.85)	−0.29 (−0.42 to −0.10)
<20	>30	22	1.33 (0.89–1.96)	0.75 (0.51 to 1.12)	−0.06 (−0.11 to 0.03)
					−0.35 (−0.53 to −0.07)

**Table 5:** Estimate of reduction in stroke incidence rate in the KSA by raising minimum 25(OH)D concentration to 30 ng/mL using 25(OH)D data from [86] and results from a U.S. study [84].

Lower 25(OH)D (ng/mL)	Upper 25(OH)D (ng/mL)	% in Lower Range in KSA	HR (95% CI)	1/HR (95% CI)	Stoke Reduction in the KSA (95% CI)
<20	20–30	76	1.85 (1.17–2.93)	0.54 (0.34 to 0.85)	−0.35 (−0.50 to −0.11)
<20	>30	20	1.33 (0.89–1.96)	0.75 (0.51 to 1.12)	−0.05 (−0.10 to 0.02)
					−0.40 (−0.60 to −0.09)

**Table 6:** Estimate of reduction in CVD mortality rate in the UAE by raising minimum 25(OH)D concentration to 30 ng/mL using 25(OH)D data from [85] and results from northern Europe [78].

Lower 25(OH)D (ng/mL)	% in Lower Range in UAE	HR (95% CI)	1/HR (95% CI)	Reduction for 25(OH)D 30–40 ng/mL (95% CI)	Reduction for 30–40 ng/mL (Fraction) (95% CI)
<12	31	2.21 (1.50–3.26)	0.45 (0.30–0.67)	−0.55 (−0.70 to −0.33)	−0.17 (−0.22 to −0.10)
12–16	14	1.61 (1.46–1.77)	0.62 (0.56–0.68)	−0.38 (−0.44 to −0.32)	−0.05 (−0.06 to −0.04)
16–20	12	1.65 (1.39–1.97)	0.61 (0.51–0.72)	−0.39 (−0.49 to −0.28)	−0.05 (−0.06 to −0.03)
20–30	20	1.37 (1.12–1.67)	0.73 (0.60–0.89)	−0.27 (−0.40 to −0.11)	−0.05 (−0.08 to −0.02)
>30		1.0	1.00	0	
				sum	−0.32 (−0.40 to −0.19)

**Table 7:** Estimate of reduction in CVD mortality rate in the KSA by raising minimum 25(OH)D concentration to 30 ng/mL using 25(OH)D data from [86] and results from northern Europe [78].

Lower 25(OH)D (ng/mL)	% in Lower Range in KSA	HR (95% CI)	1/HR (95% CI)	Reduction for 25(OH)D 30–40 ng/mL (95% CI)	Reduction for 30–40 ng/mL (Fraction) (95% CI)
<12	17	2.21 (1.50–3.26)	0.45 (0.30–0.67)	−0.55 (−0.70 to −0.33)	−0.09 (−0.12 to −0.06)
12–16	32?	1.61 (1.46–1.77)	0.62 (0.56–0.68)	−0.38 (−0.44 to −0.32)	−0.12 (−0.14 to −0.09)
16–20	27?	1.65 (1.39–1.97)	0.61 (0.51–0.72)	−0.39 (−0.49 to −0.28)	−0.11 (−0.13 to −0.08)
20–30	20	1.37 (1.12–1.67)	0.73 (0.60–0.89)	−0.27 (−0.40 to −0.11)	−0.05 (−0.08 to −0.02)
>30		1.0	1.00	0	
				sum	−0.37 (−0.47 to −0.25)

**Table 8:** Estimate of reduction in CVD mortality rate in the UAE by raising minimum 25(OH)D concentration to 30 ng/mL using 25(OH)D data from [85] and CVD results from the UK [79].

Lower 25(OH)D (Median, IQR) (nmol/L)	% Pop UAE	HR (95% CI)	HR/0.54 (95% CI)	1/(HR/0.54) (95% CI)	Reduction for 25(OH)D >76.6 nmol/L (95% CI)	DeathFractionPrevented for 25(OH) >76.6 nmol/L (95% CI)
10–22.7	15	1.0	1.85	0.54 (0.44–0.67)	−0.45 (−0.56 to −0.33)	−0.07 (−0.08 to −0.05)
22.8–29.7	15	0.82 (0.68–0.98)	1.52 (1.81–1.26)	0.66 (0.79–0.55)	−0.34 (−0.45 to −0.21)	−0.05 (−0.07 to −0.03)
29.8–53.0	31	0.65 (0.54–0.79)	1.20 (1.46–1.00)	0.83 (1.00–0.68)	0.17 (−0.32 to 0)	−0.05 (−0.10 to 0.0)
53.1–76.6	20	0.54 (0.44–0.67)	1.00	0		
76.7–100+	18	0.54 (0.44–0.67)				
						−0.17 (−0.25 to −0.08)

**Table 9:** Estimate of reduction in CVD mortality rate in the KSA by raising minimum 25(OH)D concentration to 30 ng/mL using 25(OH)D data from [86] and CVD results from the UK [79].

Lower 25(OH)D (Median, IQR) (nmol/L)	% Pop KSA	HR (95% CI)	HR/0.54 (95% CI)	1/(HR/0.54) (95% CI)	Reduction for 25(OH)D >76.6 nmol/L (95% CI)	Death Fraction Prevented for 25(OH) > 76.6 nmol/L (95% CI)
10–22.7	17	1.0	1.85	0.54 (0.44–0.67)	−0.45 (−0.56 to −0.33)	−0.08 (−0.10 to −0.06)
22.8–29.7	32	0.82 (0.68–0.98)	1.52 (1.81–1.26)	0.66 (0.79–0.55)	−0.34 (−0.45 to −0.21)	−0.11 (−0.14 to −0.07)
29.8–53.0	27	0.65 (0.54–0.79)	1.20 (1.46–1.00)	0.83 (1.00–0.68)	0.17 (−0.32 to 0)	−0.05 (−0.09 to 0.0)
53.1–76.6	20	0.54 (0.44–0.67)	1.00	0		
76.7–100+	4	0.54 (0.44–0.67)				
						−0.24 (−0.33 to −0.24)

**Table 10:** Mechanisms by which vitamin D deficiency increases the risk of AD.

Factor	Reference	Mechanisms
Brain aging	[124]	[125]
Cellular senescence	[126]	[127]
Low HDL	[57]	[57]
Mitochondrial dysfunction	[124,128,129]	[130]
Oxidative stress	[131]	[130,132,133,134]

HDL, high-density lipoprotein.

## Data Availability

Not applicable.
